# Systematic identification of trimethoprim metabolites in lettuce

**DOI:** 10.1007/s00216-022-03943-6

**Published:** 2022-02-09

**Authors:** Đorđe Tadić, Michal Gramblicka, Robert Mistrik, Josep Maria Bayona

**Affiliations:** 1 Department of Environmental Chemistry, Institute of Environmental Assessment and Water Research, Spanish Council for Scientific Research (IDAEA-CSIC), Jordi Girona 18-26, 08034 Barcelona, Spain; 2 HighChem Ltd., Leškova 11, 811 04 Bratislava, Slovakia; 3 Department of Chemical and Biochemical Engineering, Faculty of Chemical and Food Technology, Slovak University of Technology, Radlinského 9, 812 37 Bratislava, Slovakia

**Keywords:** High-resolution mass spectrometry, Non-target screening, Plant metabolites, Antibiotics, Conjugates

## Abstract

Antibiotics are some of the most widely used drugs. Their release in the environment is of great concern since their consumption is a major factor for antibiotic resistance, one of the most important threats to human health. Their occurrence and fate in agricultural systems have been extensively investigated in recent years. Yet whilst their biotic and abiotic degradation pathways have been thoroughly researched, their biotransformation pathways in plants are less understood, such as in case of trimethoprim. Although trimethoprim has been reported in the environment, its fate in higher plants still remains unknown. A bench-scale experiment was performed and 30 trimethoprim metabolites were identified in lettuce (*Lactuca sativa* L.), of which 5 belong to phase I and 25 to phase II. Data mining yielded a list of 1018 ions as possible metabolite candidates, which was filtered to a final list of 87 candidates. Molecular structures were assigned for 19 compounds, including 14 TMP metabolites reported for the first time. Alongside well-known biotransformation pathways in plants, additional novel pathways were suggested, namely, conjugation with sesquiterpene lactones, and abscisic acid as a part of phase II of plant metabolism. The results obtained offer insight into the variety of phase II conjugates and may serve as a guideline for studying the metabolization of other chemicals that share a similar molecular structure or functional groups with trimethoprim. Finally, the toxicity and potential contribution of the identified metabolites to the selective pressure on antibiotic resistance genes and bacterial communities via residual antimicrobial activity were evaluated.

**Supplementary Information:**

The online version contains supplementary material available at 10.1007/s00216-022-03943-6.

## Introduction

Antibiotics (ABs) are one of the most widely used categories of pharmaceuticals [1]. ABs are used extensively in human and veterinary medicine, including aquaculture, to prevent or treat microbial infections [2]. AB consumption is a primary driver of AB resistance, the ability of microbes to evolve and withstand the effects of ABs, which is considered one of the greatest threats to human health worldwide [3, 4]. Human population growth and the intensification of food-animal production are increasing antibiotic consumption around the world [1], mostly through misuse and overuse of ABs in agriculture [5]. The COVID-19 pandemic has contributed to overuse in human medication, too, e.g. 72% of COVID-19 patients in the USA received ABs even though they were not clinically indicated [6]. Hence, there is a continual discharge of ABs into ecosystems due to animal and human application and AB manufacturing plant [7] and hospital [8] discharges.

Reclaimed wastewater, biosolids, and manure usage in agricultural practices are considered as the main input of ABs in agricultural systems, since ABs are frequently detected in these matrices [9–11]. Consequently, AB uptake by plants will occur [5, 12]. Once taken up by plants, ABs, like other xenobiotics, undergo metabolization, which can be divided into three phases: transformation, conjugation, and internal compartmentation [13]. More specifically, phase I transformations, such as hydrolysis or oxidation, activate the molecule through the addition or exposure of functional groups, rendering it suitable for further modification in phase II. However, as ABs already have functional groups, the parent compound may be directly modified in phase II, without prior transformation. In phase II, xenobiotics or their activated phase I metabolites undergo conjugation with endogenous plant compounds [14]. Conjugation of xenobiotics in plants may happen with various substituents, such as acetyl, methyl, and sulfate groups, glucuronic acid, cladinose, glucose, glutathione, glucopyranosyloxy and malonyl groups, pterin, methyl salicylate, leucyl, glutamic acid, and glutamine molecules, amongst others [15–19]. As a result, phase II might yield “endless” conjugation possibilities in plants as a detoxification mechanism. Nevertheless, the extent of phase II conjugation in plants is largely unknown. In this regard, Hurtado et al. [20] estimated that the glycosylated conjugate fraction of several contaminants of emerging concern accounted for 27 to 83% of the free parent compound. The main concern related to phase II plant metabolites is that they may easily become deconjugated, releasing the parent bioactive molecule with its full activity [19].

The 5-[(3,4,5-trimethoxyphenyl)methyl]pyrimidine-2,4-diamine (trimethoprim, TMP) has been widely used since the early 1970s, especially in combination with sulfamethoxazole, due to its efficacy in the treatment of clinical infections [21]. TMP has been detected in raw wastewaters [22], sewage sludge [23], aquatic environments (e.g. wastewater treatment plant effluents and surface waters) [1, 24], and manure [25]. Its occurrence has also been reported in vegetables [26–29], as the occurrence of its metabolite keto-TMP, which was detected in lettuce at concentrations up to 12 times higher than the parent TMP [29]. However, its metabolization in higher plants has not yet been addressed. As consequence, the screening of ABs in edible vegetables usually targets parent compounds but neglects transformation products and metabolites. This study bridges the knowledge gap in the occurrence of TMP biotransformation products in plants using lettuce as a model. These compounds, including some which are pharmacologically active, have always been overlooked, as it was their potential contribution to the selective pressure and antibiotic resistance dissemination. Indeed, only a few studies on the metabolization of sulfonamides (i.e. sulfamethoxazole, sulfadiazine, and sulfamethazine) [15–17, 30], clarithromycin [16], and ofloxacin [31] in plants have been reported. For example, clarithromycin follows four major metabolic pathways in lettuce, including cladinose hydrolysis, demethylation, methylation, and oxidation [16]. On the other hand, the main transformation pathways reported for sulphonamides are oxidation, hydroxylation, and desulfation during phase I, and conjugation with glucose, N4-glycosyl-glycoside, pterin, methylsalicylate glutathione, glucuronic acid, and leucine during phase II [15–17, 30]. These studies show that in spite of some common phase I metabolites, AB metabolization in plants is rather diverse and unpredictable. Hence, there is a necessity to identify possible biotransformation products of TMP in plants, which will serve as a basis for future target screening in order to assess comprehensive health risks. Despite the advances in instrumentation and the development of more efficient identification strategies, transformation product identification remains a tedious and time-consuming task [32]. More than ever, the implementation of advanced data analysis strategies had become essential to elucidate profiles and extract new knowledge [33]. Identification is particularly challenging when it comes to new contaminants, such as metabolites, due to their absence in spectral databases. In silico prediction of metabolization can facilitate this task; however, it cannot be used as a stand-alone tool in the assessment of the novel biotransformation pathways. To ensure comprehensiveness, the study applies exhaustive data analysis based on the combination of differential analysis (control vs exposed) and advanced spectra-processing tool, i.e., identification of ions related to in silico predicted fragments of TMP).

Notwithstanding, strategies applied for structural elucidation of unknown compounds are often missing multistage fragmentation (MS^n^). Ultimate generation of mass spectrometry instruments, such as the Orbitrap Fusion Lumos Tribrid Mass Spectrometer used in this study, provides MS^n^ spectra which substantially increase the confidence in the identification of complex compounds, e.g. phase II metabolites. The applicability of the Orbitrap Mass Spectrometer has been already confirmed [31]; moreover, Bade et al. [34] observed slightly better performances of Orbitrap compared with QTOF, although the non-target screening results were very similar. In light of these considerations, the main aim of this study is to identify the TMP metabolites in lettuce (*Lactuca sativa* L.) as a model plant, using high-resolution LC-HRMS^n^ spectra with an emphasis on the unknown phase II metabolites (plant conjugates), and to suggest the major biotransformation reactions.

## Material and methods

### Chemicals

Methanol, acetonitrile, and water (LC–MS grade) were purchased from Sigma-Aldrich (St. Louis, MO, USA), as was the formic acid (98–100%, pro analysis). TMP was purchased from Sigma-Aldrich, whilst the keto-TMP (CAS 30806–86-1) and hydroxy TMP (CAS 29606–06-2) were purchased from LGC standards S.L.U. (Barcelona, Spain). All standards were high-purity (95% or higher).

### Experimental setup and sample extraction; chromatographic and mass spectrometry conditions

The same experimental setup was used as previously described [31, 35]. Briefly, 1 g of lettuce (*Lactuca sativa* L.) seeds was spread over the plastic tray to germinate and the seedlings were grown in a hydroponic system in Hoagland’s nutrient solution. Exposed lettuce samples were irrigated with nutrient solution fortified with TMP (5 µg mL^−1^), whilst control samples were grown in absence of TMP. Plants were grown in triplicates and were harvested after 30 days. Roots and leaves were separated, stored at − 20 °C until analysis, and analysed separately. Ultrasound-assisted extraction (35 kHz) was performed in two cycles of 15 min applying methanol (2 × 10 mL) as an extraction solvent. The sample mass was 1 g of the fresh weight. Supernatants were combined and evaporated under a gentle stream of nitrogen until the volume had been reduced to 1 mL. After dilution with 10 mL water, extracts were percolated through Strata-X cartridge (100 mg sorbent, 6 cc) from Phenomenex (Torrance, CA, USA). The cartridges were previously preconditioned with 6 mL of methanol and 6 mL of water. Two millilitres of elution solvent, a mixture of methanol and ethyl acetate (1:1, v:v), was applied. The eluted fraction was evaporated to dryness, reconstituted in water, and filtered through 0.22-µm PTFE membranes (Frisenette, Knebel, Denmark) shortly before injection to the ultra-high-performance liquid chromatography (UHPLC).

The UltiMate 3000 UHPLC station (Dionex, Thermo Fisher Scientific, Waltham, MA, USA) was equipped with a Kinetex C18 column (particle size 2.6 µm, ID 2.1 mm, length 50 mm; Phenomenex, Torrance, CA, USA), including a pre-column (2.1 mm × 5 mm) containing the same packing material. The Orbitrap Fusion Lumos mass spectrometer (Thermo Fisher Scientific, Waltham, MA, USA) was equipped with heated electrospray ionization (H-ESI). The full scan (MS^1^) was performed using an Orbitrap detector at a resolution of 60,000, in the mass range from 50 to 1000 Da. External mass calibration was frequently performed (prior to each sequence) using reference mixture of compounds with a well-known m/z values; hence, an adequate accuracy of less than 5 ppm was obtained. To obtain the MS^2^ and MS^3^ scans, two fragmentation techniques at different collision energies were performed; hence, poor reproducibility was avoided, namely, collision-induced dissociation (CID) at normalized collision energies of 20, 25, 40, and 60 and higher-energy collisional dissociation (HCD) at normalized collision energies of 20, 30, and 60. More information can be found elsewhere [31].

### Data processing, identification, and confirmation

High-resolution chromatograms were processed using Xcalibur™ 4.1 software (Thermo Fisher Scientific, Waltham, MA, USA) with a 5 ppm mass tolerance. Fragment Ion Search (FISh) and MS/MS fragmentation prediction, both features of the Mass Frontier™ 8.0 software (HighChem, Bratislava, Slovakia), were used to extract peaks related to the parent TMP in metabolites and to simulate compounds’ fragment ions, respectively, which were compared with the fragmentation information from the samples. The EPI (Estimation Programs Interface) Suite™ was used to estimate the physical–chemical properties of the identified compounds [36]. Chemicalize (developed by ChemAxon) was used for the prediction of log *D* [37]. The ecological structure–activity relationships, or ECOSAR, predictive model [38] was used to estimate the acute (short-term) and chronic (long-term or delayed) toxicity of the identified metabolites to aquatic organisms such as fish, aquatic invertebrates, and aquatic plants. As previously described [31], two approaches were used to compile the list of possible TMP metabolites after the initial MS^1^ full-scan acquisition. The first was an in-house developed algorithm that compared the mass chromatograms (MS^1^ full scans only) of the exposed samples (grown on a medium fortified with TMP) with those of the metabolite-free control samples. Ions with a peak intensity of over 70% between the exposed and control samples were targeted for further evaluation. The second approach used the abovementioned FISh tool, a feature of the Mass Frontier™ 8.0 software enabling advanced spectra-processing methods, to identify possible metabolites.

Accurately measured mass, the ring double bond (RDB) equivalent value, and comparison with the theoretical isotope pattern of the proposed sum formula were the basis for the proposed elemental composition of the detected ions. Since the adducts formed in the ion source and the charge are known, any formula giving an inappropriate RDB equivalent value should give enough evidence to reject them [39]. Spectral trees created through MS^2^ and MS^3^ acquisitions generally produce mass spectra with minimal interferences as only ions within a selected m/z window are fragmented. Thus, the structure elucidation method was based on characteristic fragmentation observed from data-dependent MS/MS and MS^n^ data. The level of confidence regarding the identification of the detected compounds was classified according to Schymanski et al. [40].

## Results and discussion

### Systematic identification of TMP metabolites

Lack of analytical standards and consequently high-resolution MS/MS spectra of antibiotic biotransformation products makes target and/or suspect screening challenging. On the other hand, compiling a list of expected compounds based on the common biotransformation reactions may be helpful but still limited. Indeed, any biotransformation product which followed a different path than previously included in our estimation would remain undetected. As it will be discussed below, phase II of the plant metabolization process includes rather a diverse group of reactions. To overcome these problems, this study applies two complementary strategies. A comparison of MS^1^ full-scan chromatograms (control vs exposed) laid the groundwork for the identification of TMP metabolites. The comparison yielded a list of 1018 ions as possible metabolite candidates. This is a comprehensive but less selective approach since the compiled list consists of TMP metabolites together with plant secondary metabolites that were released due to the exposure of the plant to the TMP. Another more specific and complementary approach was applied, namely, the advanced spectra-processing tool FISh. FISh makes it possible to selectively extract and identify metabolite peaks from the ions of the background matrix by searching for ions related to in silico predicted fragments, in this case of TMP. In combination, these two approaches enabled the selection of 87 candidates for further analysis. Spectral trees (MS^2^ and MS^3^ analysis) of these 87 ions were generated to facilitate the assignment of molecular structures to unknown compounds. Finally, 30 TMP metabolites were identified with a mass error lower than 4 ppm, 19 with identification confidence levels [40] 1 and 3 (Table 1), and 11 with a level of 4 or 5 (Table 2) illustrating the suitability of the applied method for the identification and structural elucidation of unknown molecules. Of these metabolites, three (TMP681, TMP445, and TMP553_b) were detected only in roots, whilst two (TMP325 and TMP553_a) were detected only in leaves; the rest of the metabolites were detected in both compartments. Relatively poor chromatographic separation was observed for some compounds (Table 1), especially for more hydrophobic ones due to the applied specific gradient, which was adjusted for more polar compounds [31]. Data-dependent MS/MS of the compounds with similar retention times have been thoroughly analysed and compared to exclude the possibility that they were generated from the co-eluted precursor ions. Mass spectra of all compounds and additional fragmentation pattern descriptions which are not crucial for the molecular structure assessment are presented in the Supplementary Material (Figures S1–S30).

**Table 1:**
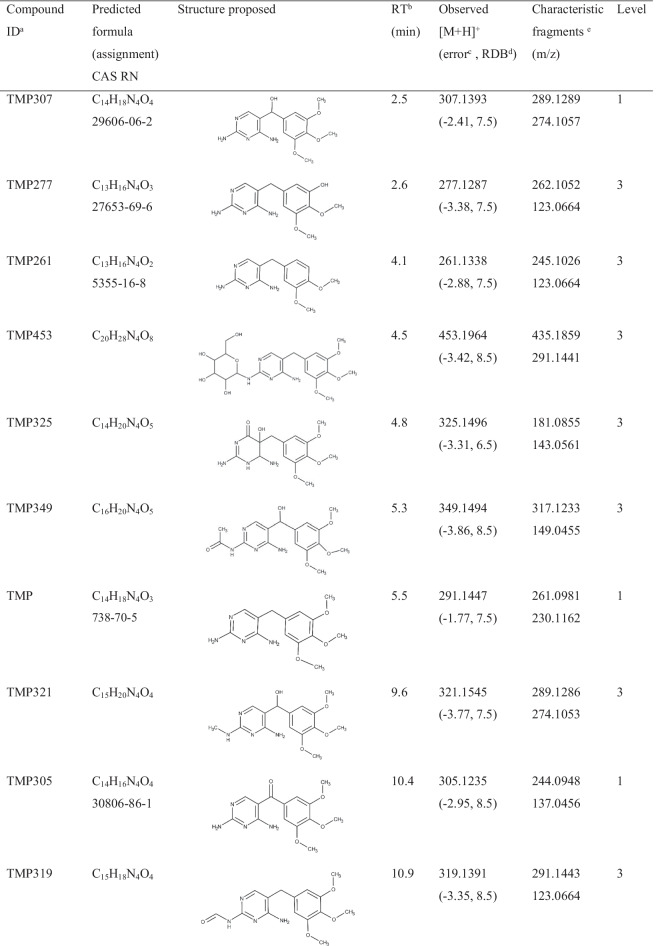

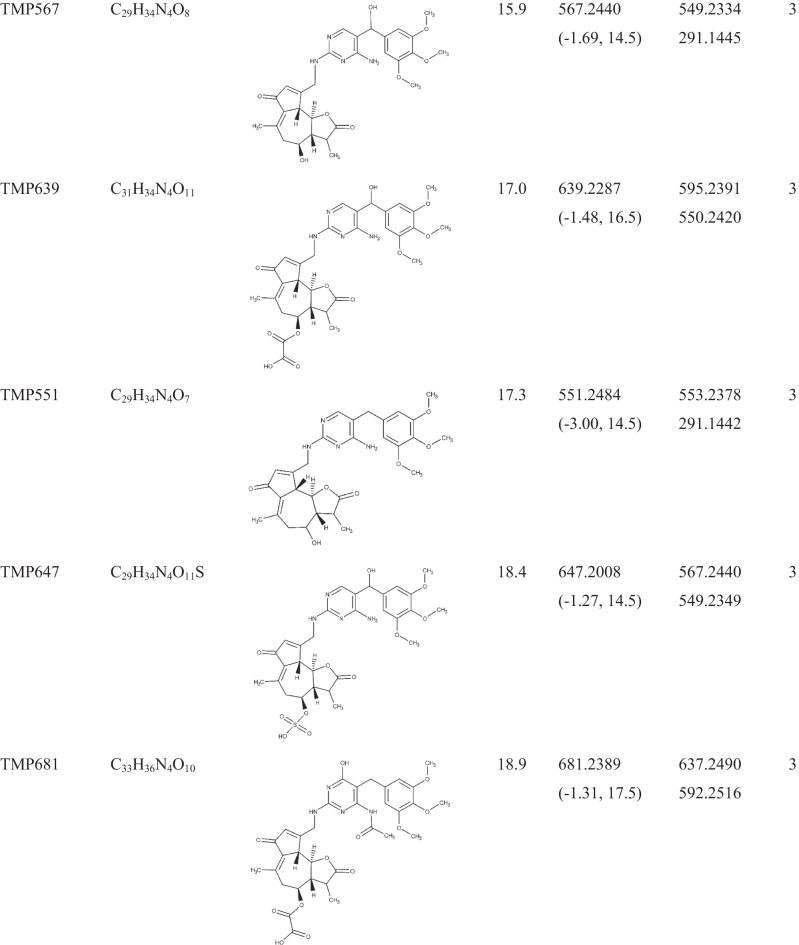

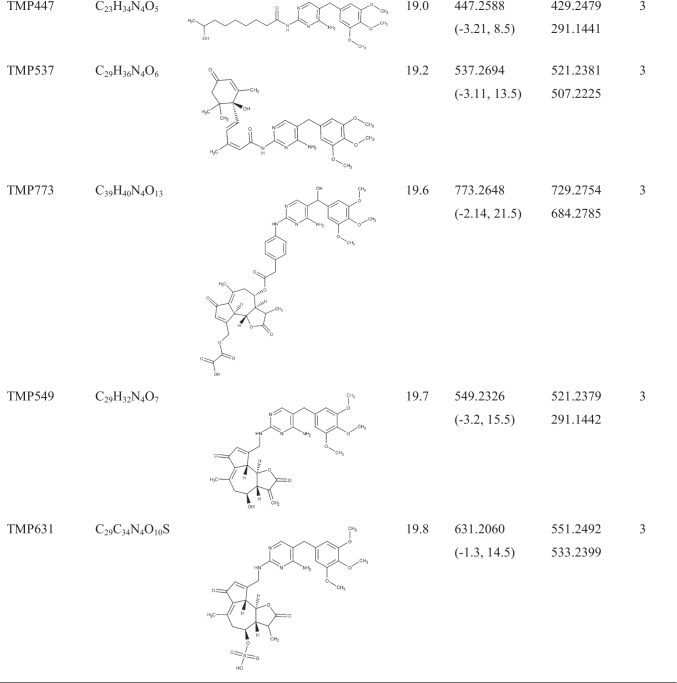
Molecular formula, structure, retention time, and mass spectral information of TMP and its metabolites identified with confidence levels 1 (structure confirmed with a reference standard) and 3 (tentative candidates)

^a^The metabolite code number is referring to the pseudo-molecular ion (M + 1). ^b^Retention time. ^c^Error is given in ppm. ^d^Ring double bond equivalent. ^e^The most abundant fragments in average CID spectrum

**Table 2:** Mass spectral information and retention time of TMP metabolites identified with confidence levels 4 (unequivocal molecular formula) and 5 (an exact mass of specific interest for the investigation)

Compound ID^a^	Molecular formula	RT^b^ (min)	Parent/exact mass	Main fragments	Level
TMP390	C_18_H_23_N_5_O_5_	4.9	390.1758	373.1492, 345.1544, 319.1388, 303.1440, 291.1441	4
TMP339	C_15_H_22_N_4_O_5_	6.1	339.1651	321.1340, 307.1391, 181.0855, 123.0439	4
TMP347	C_17_H_22_N_4_O_4_	7.9	347.1702	332.1468, 331.1390, 317.1233, 314.1363, 286.1415, 239.1285, 179.0924	4
TMP553_a		8.9	553.4444	535.4894, 371.2263, 291.1443, 263.2361, 245.2257, 183.1739	5
TMP315	C_16_H_18_N_4_O_3_	9.1	315.1440	300.1209, 299.1129, 285.0972, 282.1102, 254.1154, 134.0584	4
TMP463		10.6	463.1560	445.1455, 401.1556, 348.1906, 291.1440, 283.0931. 203.0521	5
TMP469		14.5	469.1703	439.1232, 407.1336, 363.1438, 291.1441, 262.1145, 226.0936	5
TMP553_b		17.6	553.2580	535.2446, 533.2379, 521.2016, 291.1442	5
TMP475		18.2	475.2532	457.2435, 431.2273, 355.1190, 291.1442	5
TMP517		19.1	517.2638	499.2535, 487.2171, 343.0598, 291.1441	5
TMP445		19.4	445.2343	427.2328, 415.1966, 411.2019, 369.1547, 291.1443	5

^a^The metabolite code number is referring to the pseudo-molecular ion (M + 1). ^b^Retention time

**Fig. 1:**
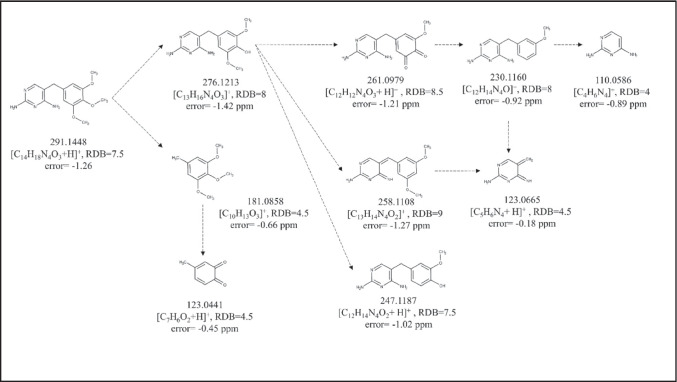
Characteristic fragmentation pathways of TMP with proposed structures for the neutral form. The main reactions included the fragmentation of methoxy groups, such as loss of CH_3_ (− 15.0235), C_2_H_6_ (− 30.0469), CH_5_O (− 33.0340), C_2_H_5_O (− 44.0469), and C_2_H_5_O_2_ (− 61.0288)

As it can be seen in Fig. 1, where the main fragments of TMP are presented, the m/z value 181.0858 indicates the trimethoxy phenyl moiety, whilst 123.0665 and 110.0586 indicate the diaminopyrimidine one. These fragments were particularly helpful for the allocation of the site on the TMP scaffold where the biotransformation took place. For **TMP307** (Figure S1) and **TMP305** (Figure S2) the mass difference between these two compounds and the parent TMP is indicating an additional hydroxy (+ 15.9954) and keto groups (+ 13.9794), thus wereassigned as α-hydroxy TMP and keto-TMP, respectively. Two TMP307 fragments revealed the position of the OH group, namely, the m/z value 139.0612 ([C_5_H_6_N_4_O + H]^+^, RDB = 4.5, error =  − 1.78 ppm), which corresponds to 1-methyl 2,4-diaminopyrimidine plus a hydroxyl group in the highly reactive dibenzylic position, and 197.0823 ([C_10_H_13_O_4_]^+^, RDB = 4.5, error = 2.25 ppm), which corresponds to 5-methyl-1,2,3-trimethoxybenzene with a hydroxyl group in thse benzylic position. Similarly, in the case of TMP305, the m/z value 195.0648 ([C_10_H_11_O_4_]^+^, RDB = 5.5, error =  − 2.28 ppm) corresponds to 5-methyl-1,2,3-trimethoxybenzene plus an oxygen, and 137.0456 ([C_5_H_5_N_4_O]^+^, RDB = 5.5, error =  − 1.44 ppm) corresponds to 1-methyl-2,4-diaminopyridine plus oxygen. Consequently, we can conclude that both hydroxyl and oxo groups are located on the reactive dibenzylic position of the parent TMP. Finally, the suggested molecular structures of both compounds were confirmed with the reference standards reaching level 1.

The most straightforward confirmation that a potential biotransformation product was related to a TMP metabolite was the presence of the m/z 291.1441 in its MS^2^ spectra since this value corresponds to TMP. Further MS^3^ analysis of 291.1441, in those cases in which the TMP fragmentation pattern presented in Fig. 1 was revealed, provided conclusive evidence of the presence of TMP in the structure of the analysed compound. However, LC-HRMS/MS did not provide sufficient information to unambiguously determine the structure of 17 compounds but their tentative structures, achieving level 3 of identification confidence (Table 1). More specifically, it was practically impossible to distinguish between structural isomers (e.g. N^2^ or N^4^ conjugates). For biotransformation products, i.e. TMP549, TMP551, TMP631, and TMP537, MS^n^ spectra provided characteristic fragments of the conjugated compound that allowed us to confirm its structure by matching them with the mass spectral databases. For instance, the estimated difference between **TMP549** (Figure S3) and TMP indicates *N*-conjugation with lactucin. This assignment was based on the fact that m/z values typical for lactucin (e.g. 259.0956, 241.0852, 231.1008, 213.0905, 185.0956, and 175.0749) [41] matched the ones observed in the TMP549 spectrum; hence, *N*-lactucin conjugate with TMP was assigned. Another sesquiterpene lactone, dihydro-lactucin, was proposed as a conjugated molecule for **TMP551** (Figure S4). Although spectra of dihydro-lactucin is not available, it was identified based on the presence of the aforementioned fragments of lactucin [41] plus the difference of 2.0156, which occurs due to the hydrogenation. An interesting fragmentation pattern was observed for **TMP631** (Figure S5), as its spectrum contains a previously described metabolite (TMP551). Namely, the mass spectrum of TMP631 revealed 551.2492 ([C_29_H_34_N_4_O_7_ + H]^+^, RDB = 14.5, error =  − 1.46 ppm), whose MS^3^ spectrum matches that described for TMP551. Hence, TMP631 compound consists of TMP551 with the addition of SO_3_. The presence of SO_3_ is based on the observed neutral loss of 79.9568 between TMP631 and its main fragment, 551.2492. In fact, a sulfate group has already been reported as possible in sesquiterpene lactone conjugates in *Lactuca* spp. [42]; thus, the assigned structure for TMP631 was *N*-dihydro-lactucin sulfate TMP. Another biotransformation compound, **TMP537** (Figure S6), was identified as *N*-abscisic TMP. A mass difference of 246.1248 compared with TMP accounts for conjugation with abscisic acid. In fact, several m/z values (e.g. 247.1322, 219.1374, 163.0751, 135.0803, and 107.0855) were observed in the TMP537 spectrum which match m/z values from the mass spectrum for abscisic acid, available in an online library [43]. Since there is no CO_2_ loss in the fragmentation, it can be concluded that the conjugation happened between the carboxylic group of abscisic acid and the amino group of TMP, with a water loss. One m/z value that supports this hypothesis is 370.1989 ([C_20_H_26_N_4_O_3_]^+^, RDB = 10.0, error =  − 2.73), as it corresponds to methyl 2,4-diaminopyrimidine with conjugated abscisic acid. **TMP277** (Figure S7) and **TMP261** (Figure S8) were assigned as a mono-demethylated and mono-demethoxylated TMP, respectively. Neither the spectral trees nor the physical–chemical properties provided enough information to distinguish the exact methoxy group on which biotransformation took place. For **TMP349** (Figure S9), two possible structures could be proposed based on the mass spectra. The detected mass suggests either the additional hydroxy and acetyl groups or conjugation with acetic acid. Given the common phase I and II reactions, it is more likely that the hydroxy TMP underwent acetylation in the metabolic process, rather than conjugation with acetic acid [16, 44, 45]; hence, it was assigned as *N*-acetyl hydroxy TMP. The occurrence of 181.0854 indicates that the 1,2,3-trimethoxybenzene remained intact during the metabolization process, whilst the occurrence of 181.0716 ([C_7_H_9_N_4_O_2_]^+^, RDB = 5.5, error =  − 2.66 ppm) indicates that the conjugation occurred on the 2,4-diaminopyrimidine, since its mass is the sum of the typical methyl 2,4-diaminopyrimidine fragment (123.0064) and the hydroxyl and acetyl groups (58.0051). In addition, the fragment 137.0455 ([C_5_H_5_N_4_O]^+^, RDB = 5.5, error =  − 2.17 ppm) corresponds to the 2,4-diaminopyrimidine part of the TMP molecule with an additional hydroxyl group. Again, the exact amino group on which biotransformation took place remained elusive. However, the effect of the position of the OH group on the molecular hydrophobicity (expressed as a log *D* value) made it possible to surmise its likeliest location. The tentatively suggested structure is thus also based on its log *D* value at pH 2.6 (mobile phase). If located on the methylene bridge, OH would decrease its hydrophobicity (log *D* =  − 1.01) compared to the hydroxylated 2,4-diaminopyrimidine (log *D* = 1.08). Due to the fact that TMP349 eluted before TMP which has log *D* =  − 0.19, the OH is more likely to be located on the methylene bridge. The estimated difference (34.0055) between **TMP325** (Figure S10) and TMP reflects the addition of one oxygen and one hydroxy group and saturation of the double bonds, which might happen on the 2,4-diaminopyrimidine ring. This conclusion is drawn from the occurrence of two dominant fragments in the TMP325 spectrum and previously published study by Eichhorn et al. [46]. Specifically, the m/z value 181.0855 indicates that the 1,2,3-trimethoxybenzene moiety of the TMP scaffold remained intact, whilst the second dominant m/z value, 143.0561 ([C_4_H_6_N_4_O_2_ + H]^+^, RDB = 3.5, error =  − 1.69 ppm), reflects the pyrimidine ring with suggested transformations.

For the following three compounds, their identification was based mostly on the observed neutral loss which matched common, already reported biotransformation reactions. The estimated difference between TMP and **TMP321** (Figure S11) corresponds to the addition of CH_2_O; hence, separate *N*-methylation and α-hydroxylation were tentatively assigned as the biotransformation reactions involved in the formation of this compound. The presence of the m/z value 181.0857 again implies that the metabolization took place on the 2,4-diaminopyrimidine. Similarly, formyl TMP was tentatively assigned for **TMP319** (Figure S12) due to a mass increase of 27.9949 compared to TMP. One fragment served as an indicator of the formyl group position, namely 151.0611 ([C_6_H_6_N_4_O + H]^+^, RDB = 5.5, error =  − 0.98 ppm), corresponding to methyl 2,4-diaminopyrimidine and an additional formyl group. Likewise, structural assessment of the **TMP453** (Figure S13), which was tentatively assigned as *N*^*4*^-glucorinated TMP, was to great extent based on the previously reported conjugation reactions. Namely, the observed neutral loss of 162.0523 in mass spectra of TMP453 is reflecting glucose addition with water loss. Although the estimated mass difference may represent any hexose, we can suggest glucose, as it was the most common sugar involved in the conjugation process [47]. On the other hand, spectral tree of **TMP447** (Figure S14) was sufficiently rich so that its structures could be tentatively assigned. Its spectrum reveals the fragmentation pattern water loss plus double bond formation, resulting in m/z 429.2479 ([C_23_H_32_N_4_O_4_ + H]^+^, RDB = 9.5, error =  − 4.05 ppm), which is typical of the presence of a hydroxyl group. Further fragmentation led to the formation of 403.2327 ([C_21_H_30_N_4_O_4_ + H]^+^, RDB = 8.5, error =  − 3.18) due to loss of C_2_H_2_, which, in turn, yielded 319.1389 ([C_15_H_18_N_4_O_4_ + H]^+^, RDB = 8.5, error =  − 3.64 ppm) with loss of C_6_H_12_. Finally, the loss of CO led to the formation of TMP (291.1441). Hence, the TMP447 conjugate was assigned *N*^*4*^-TMP-1-hydroxynonanamide.

The sequential nature of the biotransformation processes in plants has been also revealed in the spectral trees of the following compounds. Interestingly, **TMP567** (Figure S15) appeared to be included in the structure of several compounds. Its spectrum revealed two dominant m/z values, 549.2334 ([C_29_H_32_N_4_O_7_ + H]^+^, RDB = 15.5, error =  − 1.83 ppm), formed by dehydroxylation, and 291.1445, corresponding to the parent TMP. We could easily conclude that the occurrence of m/z value 549.2334 in the MS^2^ indicates the structure of the previously described TMP549; however, spectral tree, specifically MS^3^ spectrum of 549.2334, reveals fragments characteristic of dihydro-lactucin, leading us to the conclusion that hydroxy TMP was conjugated with dihydro-lactucin. In addition, as with TMP307, the occurrence of m/z values 139.0617 and 197.0823 positioned the hydroxyl group on the highly reactive dibenzylic position. Furthermore, the mass spectrum of **TMP639** (Figure S16) revealed the structure of the previously described TMP567 with its characteristic fragments. The difference between TMP567 and TMP639 reflects the addition of C_2_O_3_, which is consistent with the reported oxalate conjugates of sesquiterpene lactones [42]. The observed fragmentation also confirms it, namely, m/z value 595.2391 ([C_30_H_34_N_4_O_9_ + H]^+^, RDB = 15.5, error =  − 0.77) was formed by neutral CO_2_ loss (− 43.9897), which was further fragmented to 567.2441 ([C_29_H_34_N_4_O_8_ + H]^+^, RDB = 14.5, error =  − 0.81 ppm) by CO loss (− 27.9950) and, finally, to 550.2420 ([C_29_H_34_N_4_O_7_]^+^, RDB = 15.0, error =  − 1.45 ppm) by oxygen loss. Thus, TMP639 was assigned as hydroxy TMP conjugated with dihydro-lactucin oxalate. Again, TMP567 appeared to be part of another metabolite, **TMP647** (Figure S17). Its mass spectrum reveals the occurrence of two dominant fragments, 567.2440 and 549.2349, and a minor one (291.1452). All characteristic fragments of TMP567 can be found in the presented spectrum, whilst the difference between TMP647 and TMP567 is estimated to be 79.9570 and corresponds to an additional sulfate group. As noted, the addition of SO_3_ to the sesquiterpene lactone has been previously described [42], so *N*-dihydro-lactucin-*O*-sulfate hydroxyl TMP was assigned for TMP647. The spectral tree of **TMP681** (Figure S18) revealed TMP567 and a fragmentation reminiscent of TMP639, indicating the presence of oxalic acid. Specifically, loss of CO_2_ led to formation of 637.2490 ([C_32_H_36_N_4_O_10_ + H]^+^, RDB = 16.5, error =  − 1.4 ppm); further fragmentation led to formation of 609.2542 ([C_31_H_36_N_4_O_9_ + H]^+^, RDB = 15.5, error =  − 1.33 ppm) by CO loss and, finally, the formation of 592.2516 ([C_31_H_36_N_4_O_8_]^+^, RDB = 16, error =  − 1.98 ppm). Fragments characteristic of dihydro-lactucin were also observed. Whereas m/z value 333.1552 ([C_16_H_20_N_4_O_4_ + H]^+^, RDB = 8.5, error =  − 0.53 ppm) reveals *N*-acetyl TMP, other m/z values (e.g. 187.1114, 215.1060, 243.1008) match the ones observed in the case of TMP567. Accordingly, the assigned structure for TMP681 is dihydro-lactucin oxalate conjugated with *N*-acetyl hydroxyl TMP. Finally, **TMP773** was assigned as *N*-dihydro-lactucopicrin oxalate-α-hydroxy TMP. Figure S19 shows a similar fragmentation pattern to that observed for TMP639 and TMP681, for which the occurrence of oxalic acid was assigned: 729.2754 ([C_38_H_40_N_4_O_11_ + H]^+^, RDB = 20.5, error =  − 1.72 ppm) formed by neutral CO_2_ loss, which was further fragmented to 701.2807 ([C_37_H_40_N_4_O_10_ + H]^+^, RDB = 19.5, error =  − 1.53 ppm) by CO loss; finally, loss of oxygen led to the formation of 684.2785 ([C_37_H_40_N_4_O_9_]^+^, RDB = 20, error =  − 0.76 ppm). The occurrence of 263.1148, 262.1186, 261.1109, 244.1077, 217.1083, and 215.1065 indicates the presence of dihydro-lactucopicrin, whose mass spectrum information is available in the MoNa library [48].

Level 4 was assigned to the four metabolites presented in Table 2 as their spectra (Figures S20–S23) provided enough information to suggest an unequivocal molecular formula, but no molecular structure could be assigned. Finally, seven metabolites were identified with a confidence level of 5 (Table 2). Although their spectra (Figures S24–S30) did not provide enough information to enable higher levels of identification confidence, there is proof that the parent TMP is incorporated in their structures.

We showcased that the applied analytical methodology successfully overcomes the abovementioned database and compound complexity problems. Moreover, the degree of confidence was significantly improved for the identification of never-before-studied compounds. The advantage of MS^3^ is particularly visible when the relatively large m/z values are provided by MS^2^, as the case for several phase II metabolites. MS^3^ decreased the number of possible molecular structures for a given pseudo-molecular feature, allowing the rebuilding of the structures of these large MS^2^ fragments, and finally of unknown parent compounds with higher confidence. However, differentiation of positional isomers remains a pitfall associated with TPs identification [32], which limits confidence levels. This is an important issue when it comes to retention time prediction. As it was shown in the case of TMP349, different position of a functional group affects differently the overall polarity of a molecule. To overcome these limitations, further analysis with nuclear magnetic resonance spectroscopy alone or in combination with LC-HRMS would be required [32].

### Biotransformation reactions

**Fig. 2:**
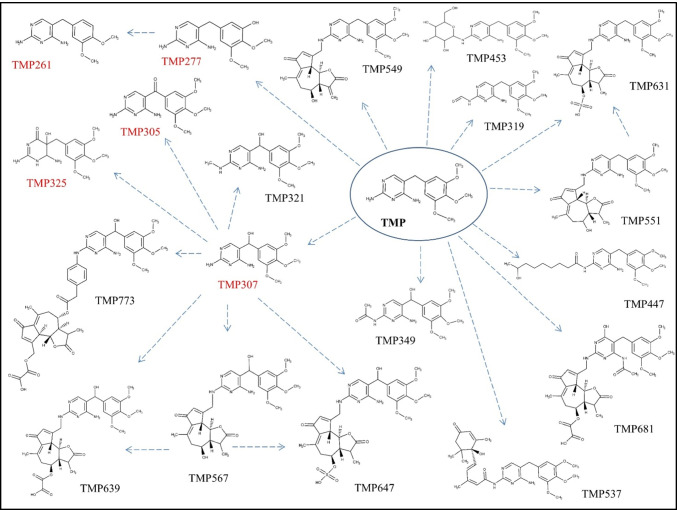
Proposed biotransformation pathways of TMP in lettuce. Metabolites labelled with red colour belong to the phase I, whilst the rest to the phase II of plant metabolism

Five phase I metabolites (TMP277, TMP261, TMP305, TMP307, and TMP325) were formed by hydroxylation, oxidation, demethylation, or dehydroxylation reactions. These reactions have also been reported during TMP degradation by nitrifying sludge bacteria [49], algae [44], and biological wastewater treatment [50]. Hydroxylation of TMP has been reported as the most prevalent reaction of biotransformation by algae [44], and during degradation in soil [51]. In addition, Kiki et al. [44] reported several other degradation products during algae treatment, which were formed mainly by TMP cleavage and thus considered phase I. As can be seen in Fig. 2, hydroxy TMP (TMP307) appeared to be the most reactive phase I metabolite in lettuce, as it continues the metabolization process further, yielding two phase I and five phase II metabolites.

This study reveals, for the first time, the occurrence of 25 phase II metabolites of TMP, of which 14 were characterized with molecular structures also for the first time. This shows that phase II reactions, i.e. conjugation with endogenous compounds, lead to the production of rather various metabolites. Direct conjugation of TMP without prior modification was expected due to the presence of active groups on the TMP scaffold, and this phenomenon was indeed observed in 6 phase II metabolites: TMP537, TMP447, TMP551, TMP631, TMP319, and TMP453. Glycosidation of ABs in plants, which yielded information on TMP453, has previously been described as the main transformation pathway for sulfamethazine and sulfamethoxazole in *A. thaliana* [17, 30] and for ofloxacin in lettuce [31]. Similarly, TMP319 and TMP349 share metabolic pathways, i.e. formylation and acetylation, respectively, with sulfadiazine in lettuce [16]. As with TMP321, methylation has been reported for clindamycin in lettuce [16]. In recent years, several studies have been published dealing with the plant conjugates of various xenobiotics, which could shed light on the fate of ABs in plants including conjugation with sugars, amino acids, a malonyl group, glutathione, glucuronic acid, sulfate, pterin, and methyl salicylate[15, 17, 30, 52–54]. For instance, two studies showed that sulfamethoxazole in *Arabidopsis thaliana*, alongside glycosylation as the main transformation pathway, underwent conjugation with glutathione and leucine after phase I transformation [15] and direct conjugation with pterin and methylsalicylate [17], which were not observed in this study. Neither it was found a conjugation with amino acids, as one of the metabolization pathways of ibuprofen in *Arabidopsis thaliana* [52]. Notwithstanding the variety of aforementioned identified possible compounds that can play a role during the phase II transformations, novel biotransformation pathways are suggested. More specifically, this is the first study to date to identify abscisic acid a well-known phytohormone [55], and sesquiterpene lactone (STL) compounds as the conjugation molecules. STLs are a large, important, and highly diverse group of compounds (*n* = 5000) [56, 57]. They have been identified in different plants belonging to the Asteraceae family [58], whilst their oxalate and sulfate derivatives have been reported in lettuce [42, 59]. In fact, STL conjugates were the major group of metabolites found in lettuce. Eight TMP metabolites were conjugated with STLs, including 6 conjugated with dihydro-lactucin (TMP631, TMP549, TMP647, TMP639, TMP567, and TMP681), one with dihydro-lactucopicrin (TMP773), and one with lactucin (TMP537). These STLs are some of the most representative in lettuce and can be found in laticifers and vacuoles, especially when produced in response to biotic stresses [57, 58]. These findings for the first time show that another group of molecules, STLs, should be included in phase II metabolization of higher plants and added to the in silico prediction reactions.

### Toxicity of AB metabolites and their possible contribution to the growing problem of antimicrobial resistance

The application of ECOSAR to the identified metabolites showed that biotransformation of TMP in lettuce led to decreased toxicity towards aqueous organisms. For all metabolites, toxicity was lower than for the parent TMP, except for TMP447, which had a slightly lower chronic value (ChV) for fish than the parent TMP (3.2 vs 3.6) (Table S1). This is consistent with findings published by Kiki et al. [44], who reported the same phenomenon of reduced toxicity of TMP metabolites during algae biotransformation. Generally speaking, plant metabolism is unlikely to yield more toxic metabolites due to the nature of the process itself as it ultimately results in the formation of less toxic compounds [13, 14].

However, another critical question related to the biotransformation of ABs must be addressed, namely, whether the plant biotransformation inactivates the antimicrobial activity or the AB metabolites retain this activity and pose selective pressure on antibiotic resistance genes and bacteria. TMP exerts antimicrobial activity by binding to bacterial dihydrofolate reductase (DHFR), and the essential moiety of TMP for binding is 2,4-diaminopyrimidine [60–63]. Hence, TMP307, TMP277, TMP261, and TMP305 could potentially contribute to the overall residual antimicrobial activity since they have an intact pyrimidine moiety and no bounded bulky substitutes that could interfere with bacterial binding DHFR. Of these, TMP307 and TMP305 also did not undergo metabolization on the 1,2,3-trimethoxybenzene moiety, which enhances the binding of TMP with DHFR [61], and are the metabolites most likely to retain antimicrobial activity. In fact, Wang et al. [64] confirmed that TMP307 (hydroxy TMP) has similar antimicrobial activity to TMP against *E. coli* (BL21(DE3)). It can thus be hypothesized that phase I biotransformation did not inactivate antimicrobial activity. On the other hand, phase II reactions took place on the pyrimidine reducing, if not completely inactivating antimicrobial activity. However, enzymes in the human (or mammalian) digestive gut have been found to play an important role in the deconjugation process [19]. In other words, deconjugation will release the parent antibiotic with its full activity. Furthermore, TMP conjugated with thiomaltose has been shown to be as effective as the parent TMP in the treatment of urinary tract infections in a mouse model [64] since the conjugated sugar enhances solubility and acts as a carrier of the TMP. As can be seen in Table S1, almost 50% of the metabolites (11 out of 19) are more soluble than the parent TMP.

## Conclusions

The applied non-target approach (chemical structures were postulated without the aid of suspect list) provided depth and a high degree of confidence in the identification of unknown complex compounds. The use of spectral trees obtained by means of LC-HRMS^n^ and advanced spectral processing tools enabled identification of 30 compounds as the TMP metabolites in lettuce, of which 5 were phase I metabolites and the remaining 25, phase II. Molecular structures were assigned for 19 compounds at confidence levels 1 and 3. For 4 more compounds, an unequivocal molecular formula was reported, and for 7 compounds, a mass of interest was reported. To date, this is the first study to investigate the fate of TMP in a higher plant, and 14 TMP metabolites were characterized with molecular structure for the first time. Non-target screening of vegetable matrix generated large complex sets of raw data, which required an exhaustive data processing approach. The applied data treatment enabled identification and characterization of the compounds that would be normally be overlooked by conventional suspected approach as their structures and metabolization pathways were unknown. These findings will help in the elucidation of unknown metabolites of the other compounds that share a similar structure and functional groups with TMP. Moreover, novel biotransformation pathways were proposed, namely, it was revealed that sesquiterpene lactones (lactucin, dihydro-lactucin, dihydro-lactucopicrin) and abscisic acid can be exploited as conjugating agents. Consequently, improving and contributing to the comprehensives of the current in silico tools for the prediction of metabolization, as a new group of plant endogenous compounds, should be added to the prediction of phase II metabolites especially for higher plants. The insight gained into the biotransformation of TMP in lettuce makes it possible to conclude that the plant biotransformation process is extremely complex, especially phase II. The results of this study call for a deeper assessment of the possible health risks of the occurrence of the identified metabolites. In the authors’ opinion, current health risk assessment approaches, which do not include metabolites but rather just parent compounds, are open to considerable dispute. Moreover, in the case of ABs, emphasis should not be placed exclusively on the possible toxicity of metabolites, but rather their residual antimicrobial activity and possible contribution to the promotion of AB resistance.

## Supplementary Information

Below is the link to the electronic supplementary material.

Supplementary file1 (DOCX 4754 KB)

## Data Availability

Not applicable.
