# Supplementary material for: Systematic identification of trimethoprim metabolites in lettuce
Source: Anal Bioanal Chem. 2022 Feb 9;414(9):3121–35. doi: 10.1007/s00216-022-03943-6 (PMC8934764; doi:10.1007/s00216-022-03943-6)
Supplement: Supplementary file 1 — Supplementary file1 (DOCX 4754 KB) [file 216_2022_3943_MOESM1_ESM.docx]

Supplementary material

**Systematic identification of trimethoprim metabolites in lettuce**

Đorđe Tadić, Michal Gramblicka, Robert Mistrik, Josep Maria Bayona


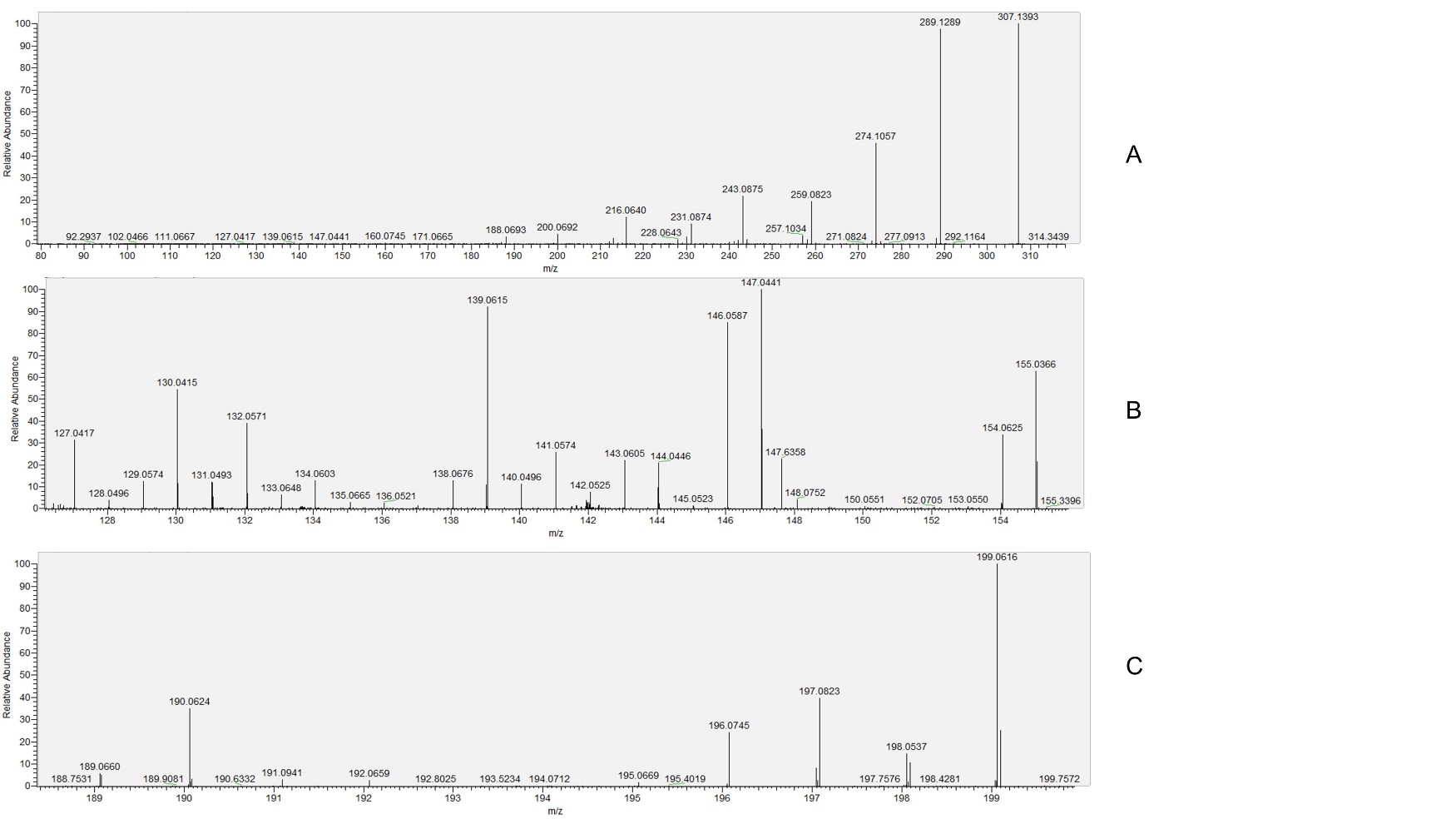


Figure S1. Mass spectrum of TMP307 (α-hydroxy TMP). A - MS^2^ CID 60eV; B and C zoomed MS^2^ CID 60eV

The most dominant fragment in the mass spectrum of TMP307 is 289.1289 ([C_14_H_16_N_4_O_3_+H]^+^, RDB= 8.5, error= -2.27 ppm), which was formed by a neutral loss of water with the formation of the double bond. It generated two additional fragments: 274.1057 ([C_13_H_14_N_4_O_3_]^+^, RDB= 9.0, error= -1.43 ppm), through loss of a methyl group, and 243.0875 ([C_12_H_11_N_4_O_2_]^+^, RDB= 9.5, error= -0.79 ppm), through loss of a methoxy group. The m/z value 259.0823 ([C_12_H_11_N_4_O_3_]^+^, RDB= 9.5, error= -1.18 ppm) is formed by demethoxylation of the TMP307 with formation of a double bond.


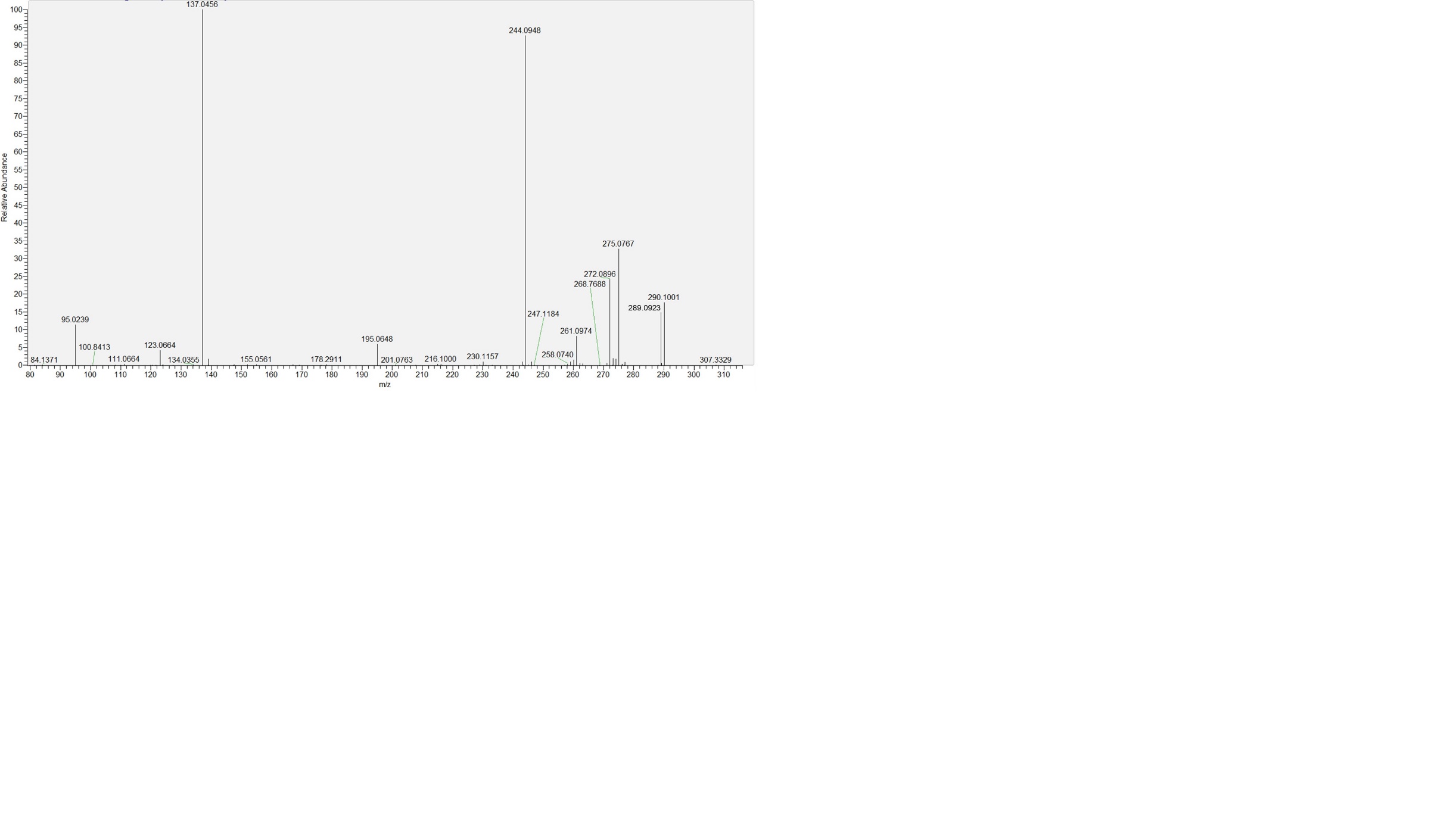


Figure S2. Mass spectrum of TMP305 (keto-TMP). (MS^2^ CID 60eV)

Fragmentation of TMP305 revealed m/z values 290.1001, 289.0924, 275.0767, and 244.0948 that correspond to characteristic fragments of TMP, plus an oxygen atom.


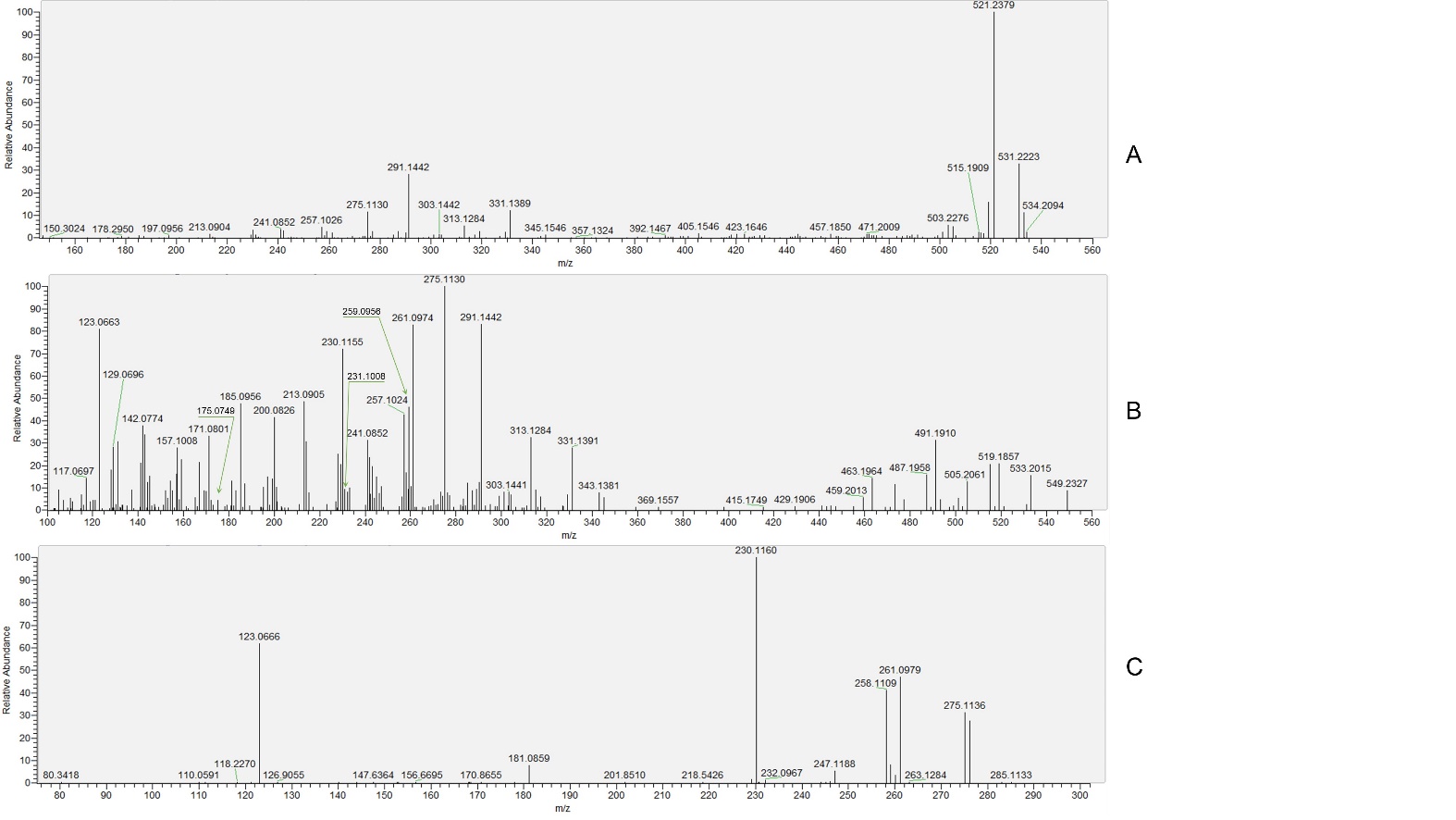


Figure S3. Mass spectrum of TMP549 (*N*-lactucin TMP). A – MS^2^ CID 60eV; B – MS^2^ HCD 60eV; C – MS^3^ 549.2326 > 291.1442 CID 60eV

The presence of TMP within the scaffold of TMP549 is clear given the occurrence of the m/z 291.1442 and the typical fragmentation pattern for TMP that can be seen in its spectrum, namely, m/z values 534.2094 (-15.0236), 533.2015 (-16.0313), 519.1857 (-30.0468), 515.1909 (-33.0338), and 291.1442. Two observed dominant fragments, 531.2223 ([C_29_H_30_N_4_O_6_+H]^+^, RDB= 16.5, error= -3.03 ppm) and 521.2379 ([C_28_H_32_N_4_O_6_+H]^+^, RDB= 14.5, error= -3.26 ppm), indicate H_2_O (-18.0104) and CO (-27.9949) loss, respectively.


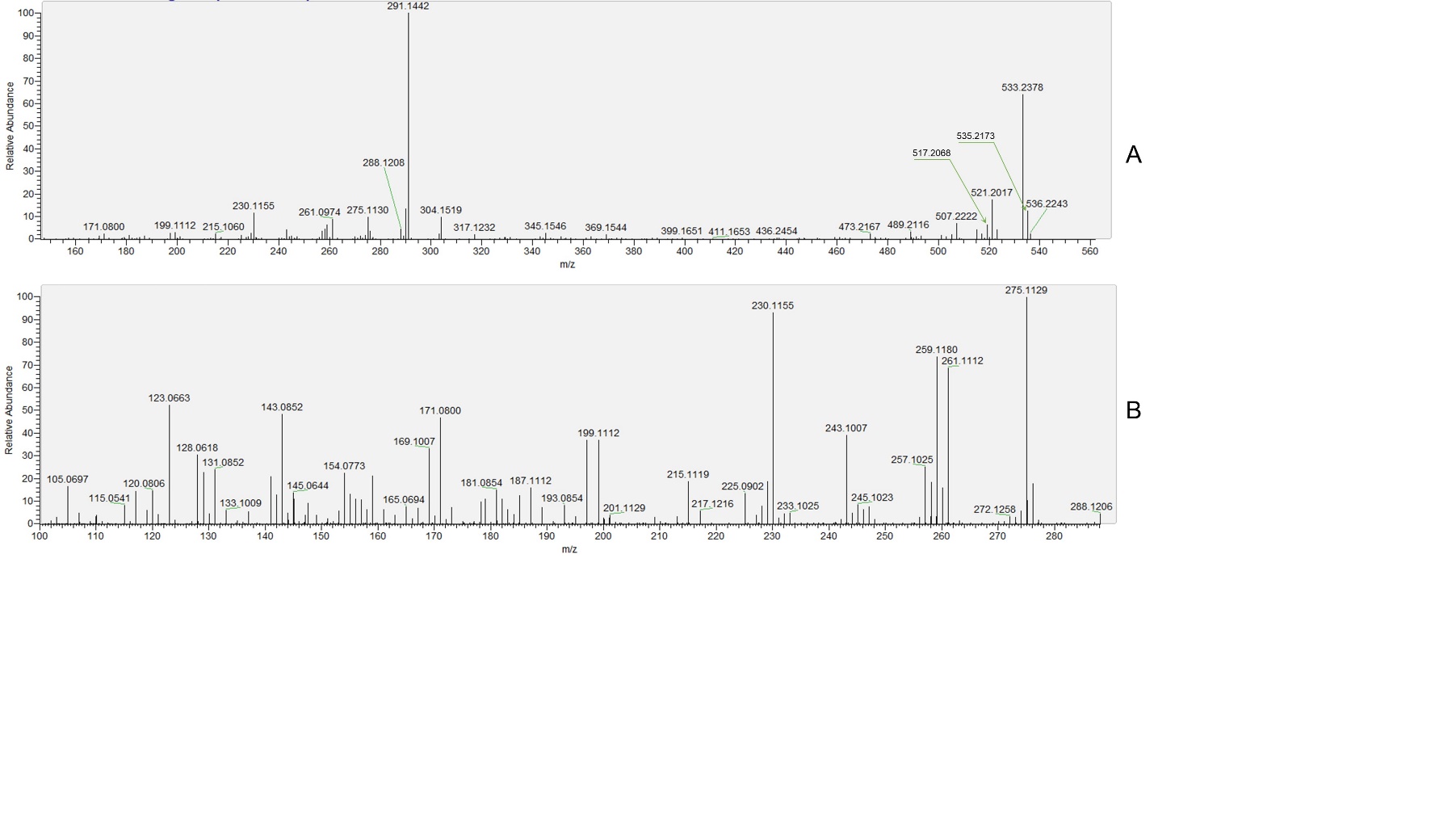


Figure S4. Mass spectrum of TMP551 (*N*-dihydro-lactucin TMP). A – MS^2^ CID 60 eV; B – MS^2^ HCD 60 eV

The mass spectrum of TMP551 showed fragmentation characteristic of TMP: 536.2243 (-15.0241), 535.2173 (-16.0312), 521.2017 (-30.0466), and 517.2068 (-34.0415). TMP presence in the metabolite was also confirmed by the occurrence of the m/z value 291.1442. Another dominant fragment, 533.2378 ([C_29_H_32_N_4_O_6_+H]^+^, RDB= 15.5, error= -3.81 ppm), was formed by dehydroxylation of TMP551.


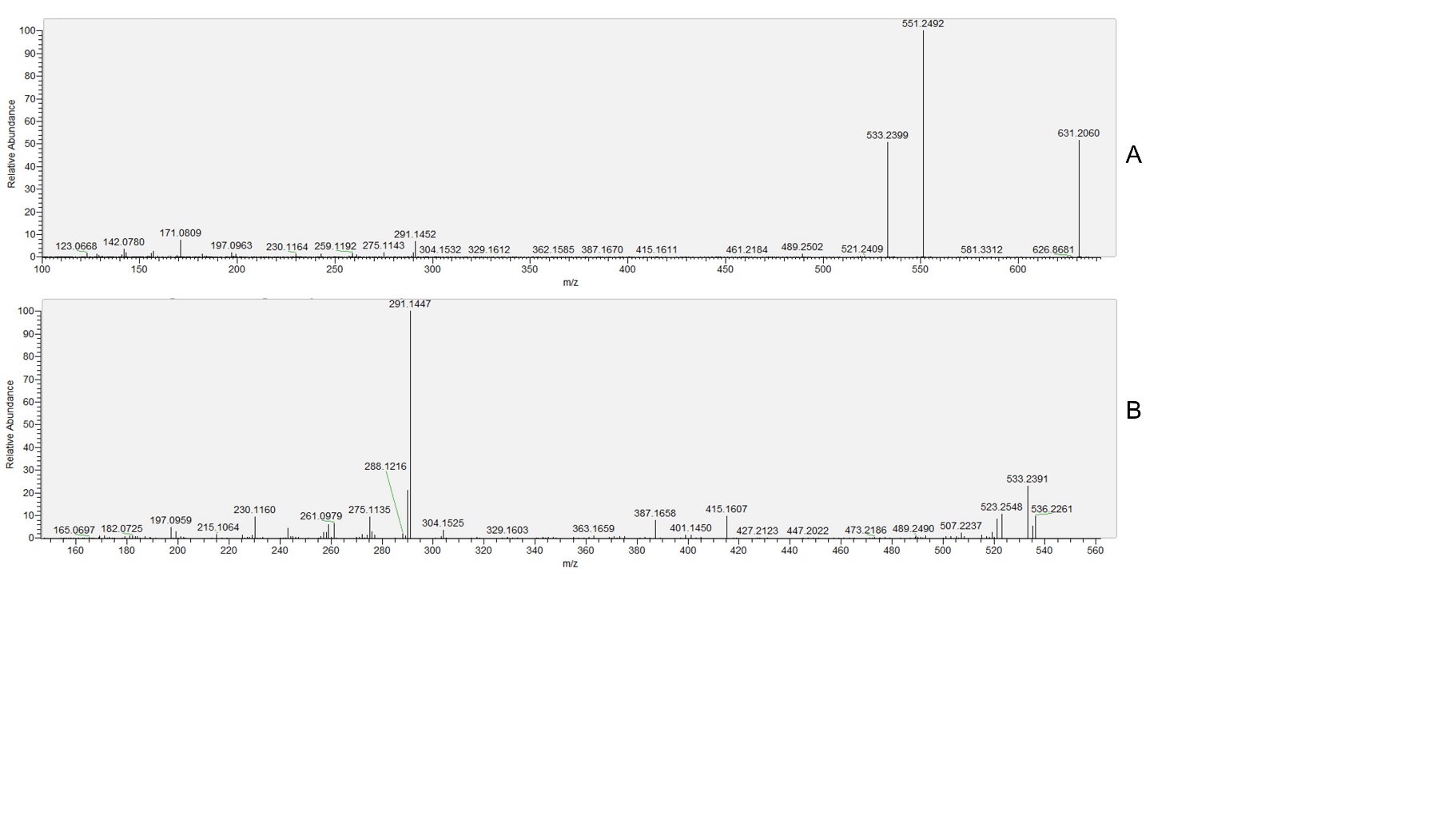


Figure S5. Mass spectrum of TMP631 (*N*-dihydro-lactucin sulfate TMP). A – MS^2^ CID 60eV; B –MS^3^ 631.2060 > 551.2492 CID 60eV


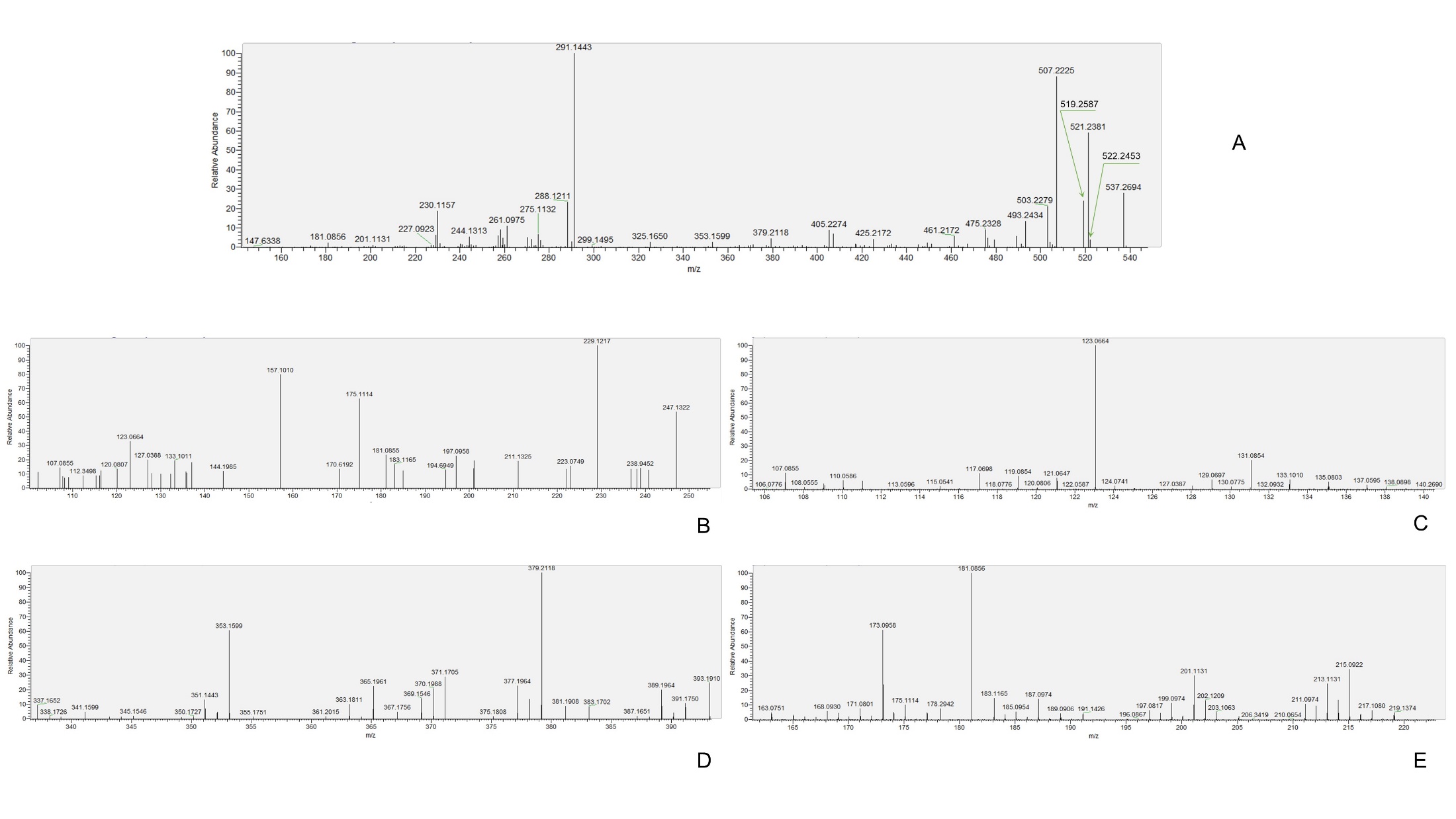


Figure S6. Mass spectrum of TMP537 (*N*-abscisic TMP). A – MS^2^ CID 60 eV; B, C, D, and E – zoomed MS^2^ CID 60 eV

Fragmentation of TMP537 revealed a pattern typical of TMP, namely, 522.2453 (-15.0238), 521.2381 (-16.0310), and 507.2225 (-30.0470), as well as the m/z value 291.1443. Another dominant fragment, 519.2587 ([C_29_H_34_N_4_O_5_^+^H]^+^, RDB= 14.5, error= -2.82 ppm), was formed by water loss, which further resulted in the formation of 503.2279 ([C_28_H_30_N_4_O_5_^+^H]^+^, RDB= 15.5, error= -2.1 ppm) by demethylation.


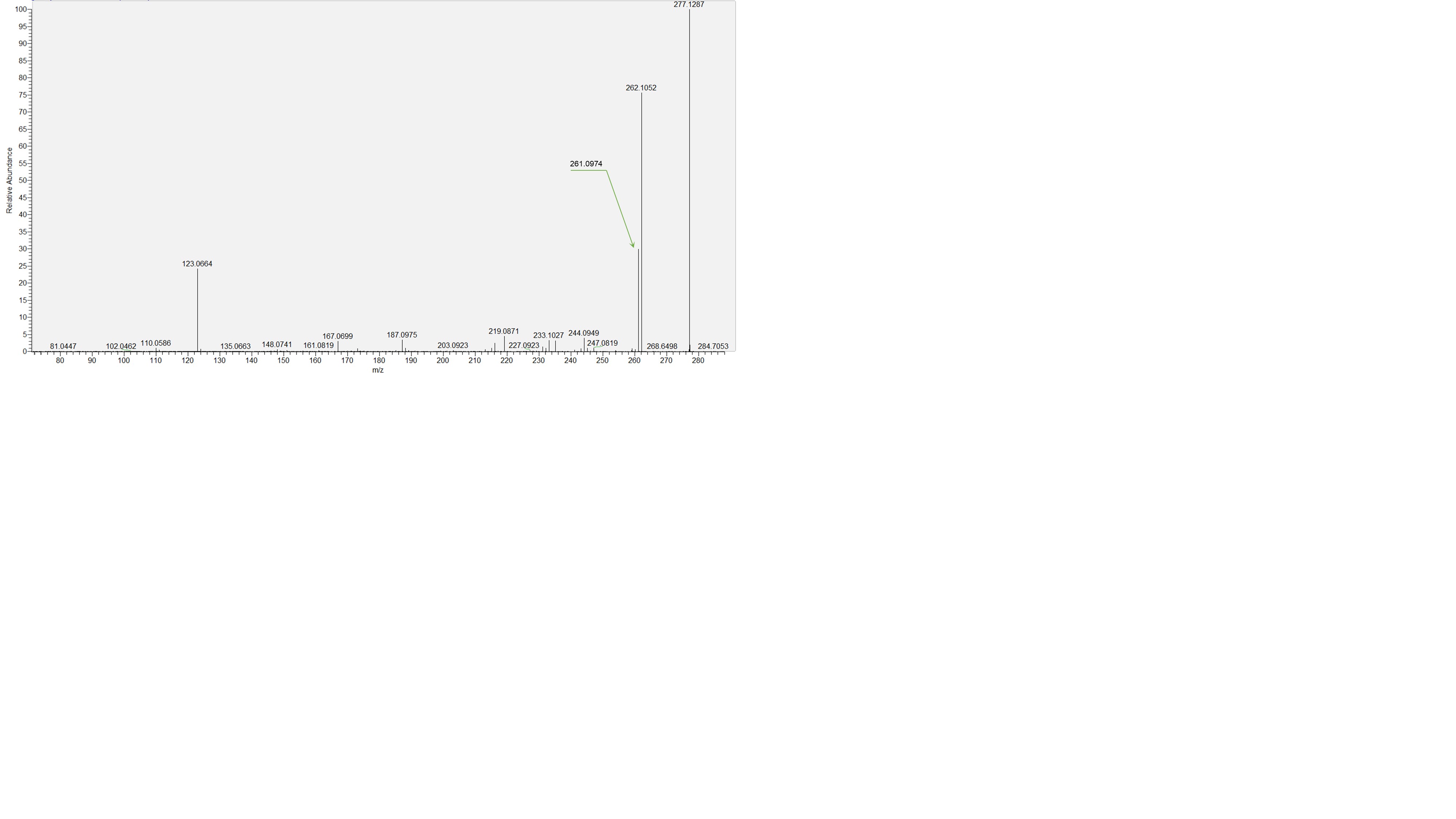


Figure S7. Mass spectrum of TMP277 (mono-demethylated TMP) (MS^2^ CID 60 eV)

Two dominant fragments of TMP277, 262.1052 ([C_12_H_14_N_4_O_3_]^+^, RDB= 8, error= -3.1 ppm) and 261.0974 ([C_12_H_13_N_4_O_3_]^+^, RDB= 8.5, error= -2.94 ppm), are formed by CH_3_ and CH_4_ loss, respectively. Similarly, 244.0949 ([C_12_H_12_N_4_O_2_]^+^, RDB= 9.0, error= -3.02 ppm) is formed by demethoxylation with double bond formation. The m/z value 167.0699 ([C_9_H_11_O_3_]^+^, RDB= 4.5, error= -2.46 ppm), accounts for half of the TMP277 molecule, namely, methylated benzene with 2 methoxy groups and one hydroxy group. In the mass spectrum of TMP277, two characteristic fragments of TMP (123.0664 and 110.0586) can be observed.


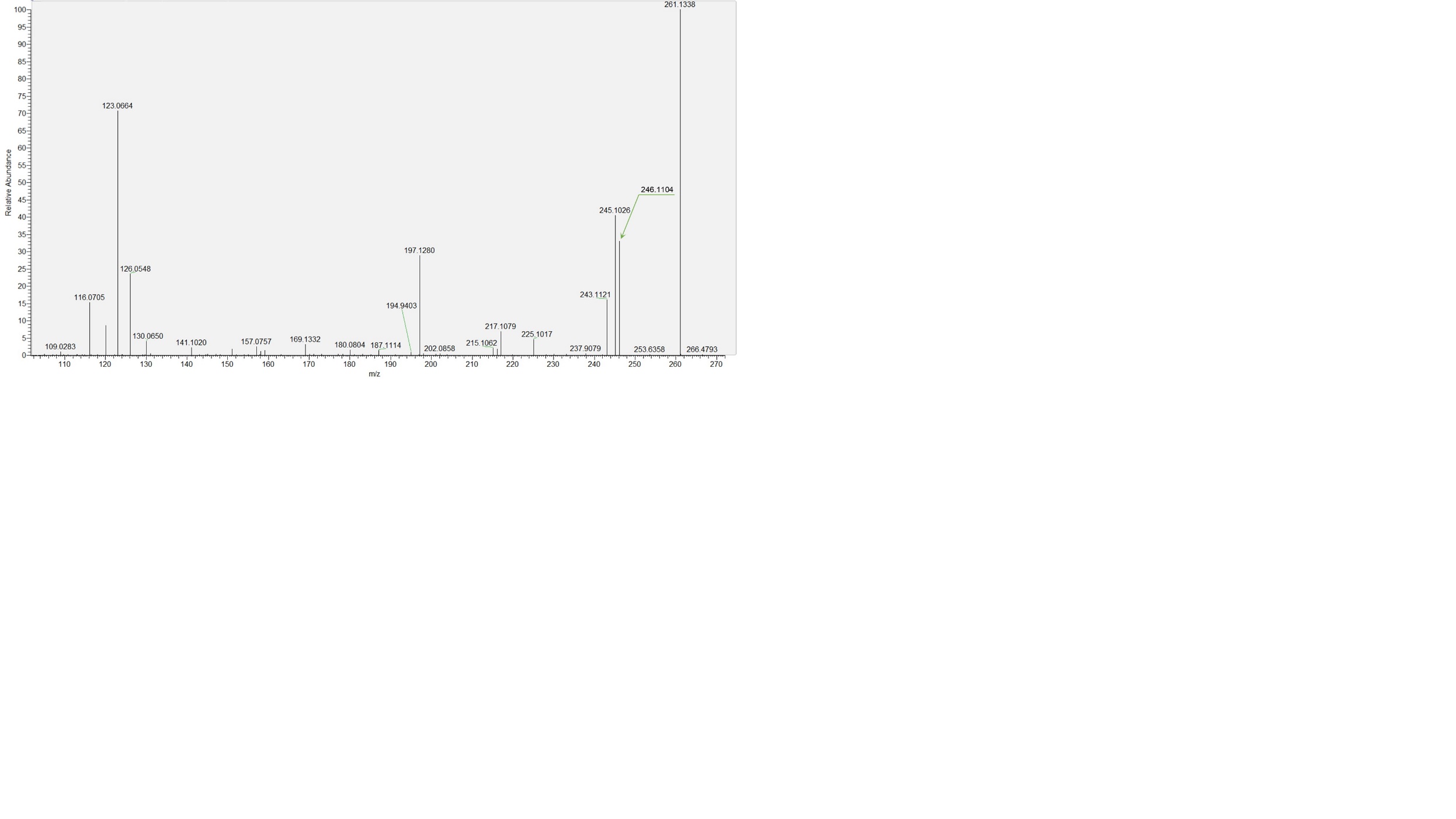


Figure S8. Mass spectrum of TMP261 (mono-demethoxylated TMP) (MS^2^ CID 60 eV)

Fragmentation of TMP261 followed the pattern of TMP, namely, the m/z value 246.1104 ([C_12_H_14_N_4_O_2_]^+^, RDB= 8, error = -2.75 ppm) is generated by CH_3_ loss and the m/z value 245.1026 ([C_12_H_13_N_4_O_2_]^+^, RDB= 8.5, error= -3.11 ppm) by loss of CH_4_. 217.1079 ([C_11_H_12_N_4_O+H]^+^, RDB= 7.5, error= -2.8 ppm) is formed by demethylation and demethoxylation. Finally, an m/z value (123.0664 ) characteristic of TMP can be observed.


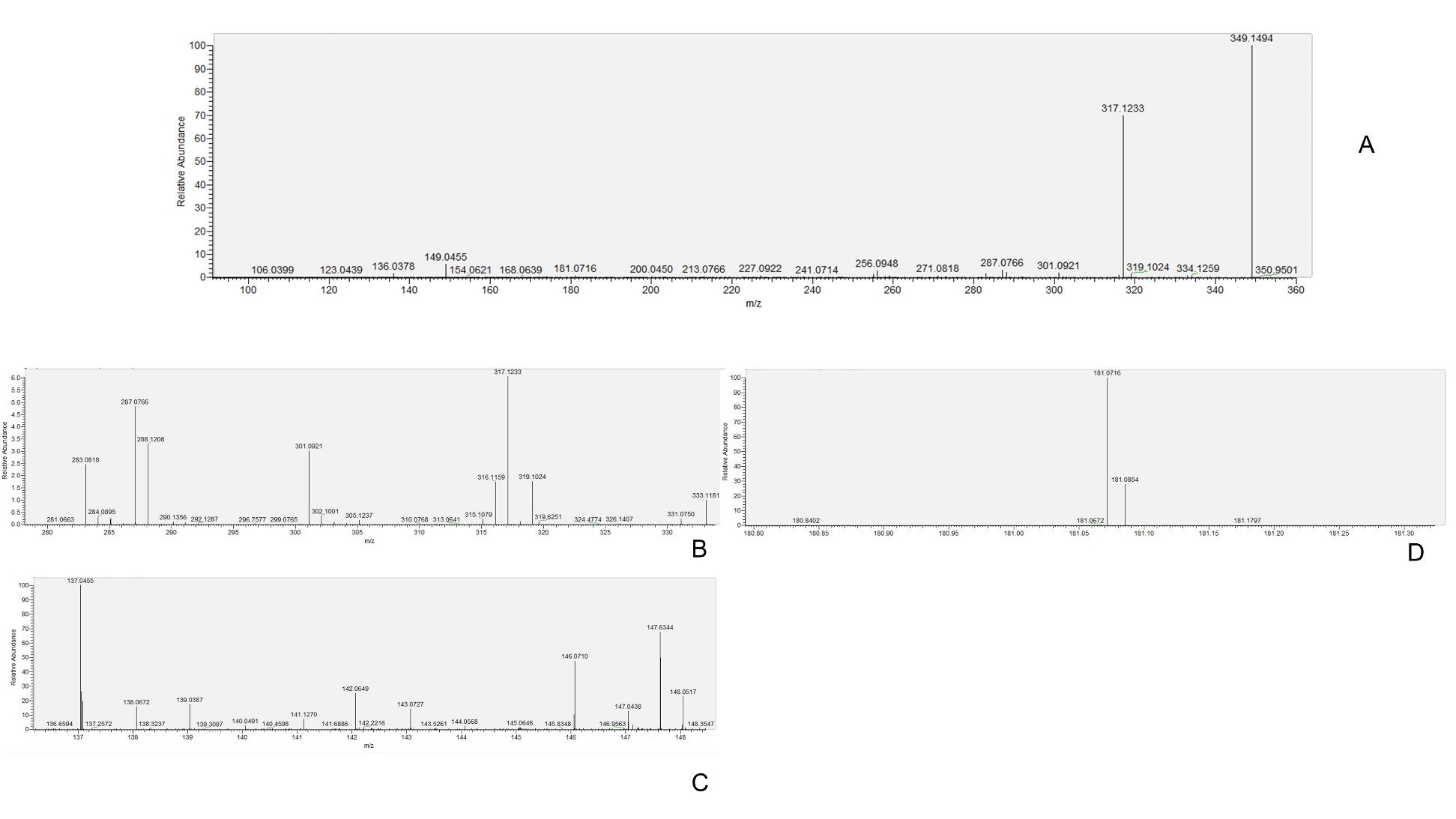


Figure S9. Mass spectrum of TMP349 (*N*-acetyl hydroxy TMP). A – MS^2^ CID 60 eV; B, C, and D – zoomed MS^2^ CID 60 eV

In the spectra of TMP349, several fragments reveal the typical fragmentation pattern of TMP: 333.1181 (-16.0313), 319.1024 (-30.0469), 316.1159 (-33.0334), and 288.1208 (-61.0285). The mass spectrum of TMP349 includes one dominant fragment, 317.1232 ([C_15_H_16_N_4_O_4_+H]^+^, RDB= 9.5, error= -3.85 ppm), formed after loss of CH_2_O.


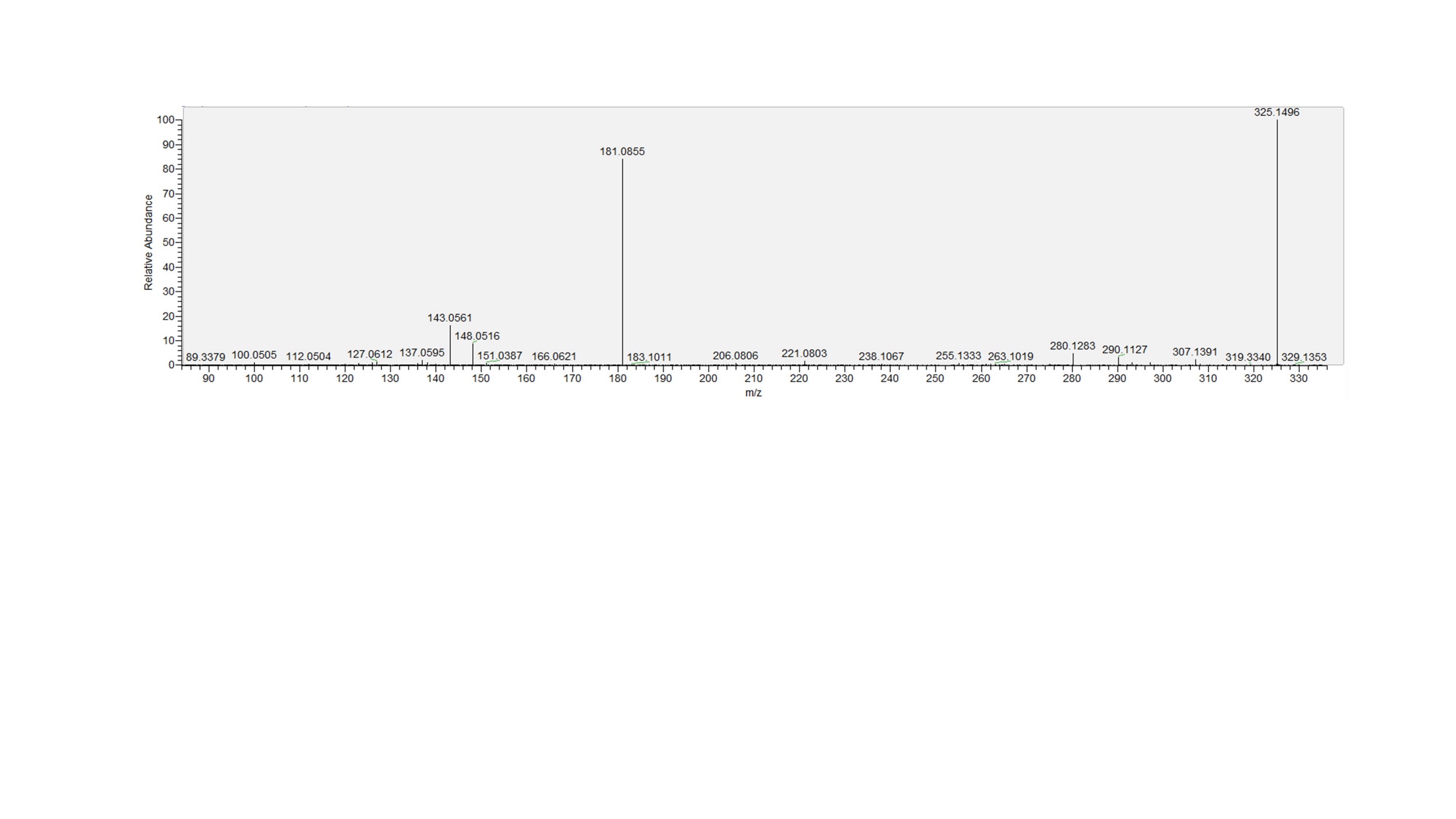


Figure S10. Mass spectrum of TMP325. (MS^2^ CID 60 eV)

In addition to the fragments explained in the main body of the manuscript, two ions observed in the spectra of TMP325 were described. Namely, m/z 307.1391 ([C_14_H_18_N_4_O_4_+H]^+^, RDB= 7.5, error= -3.16 ppm), formed by the water loss and double bond formation on the pyrimidine ring, which was further fragmented to 290.1127 ([C_14_H_15_N_3_O_4_+H]^+^, RDB= 8.5, error= -2.97 ppm) by loss of an amino group with an additional double bond formation.


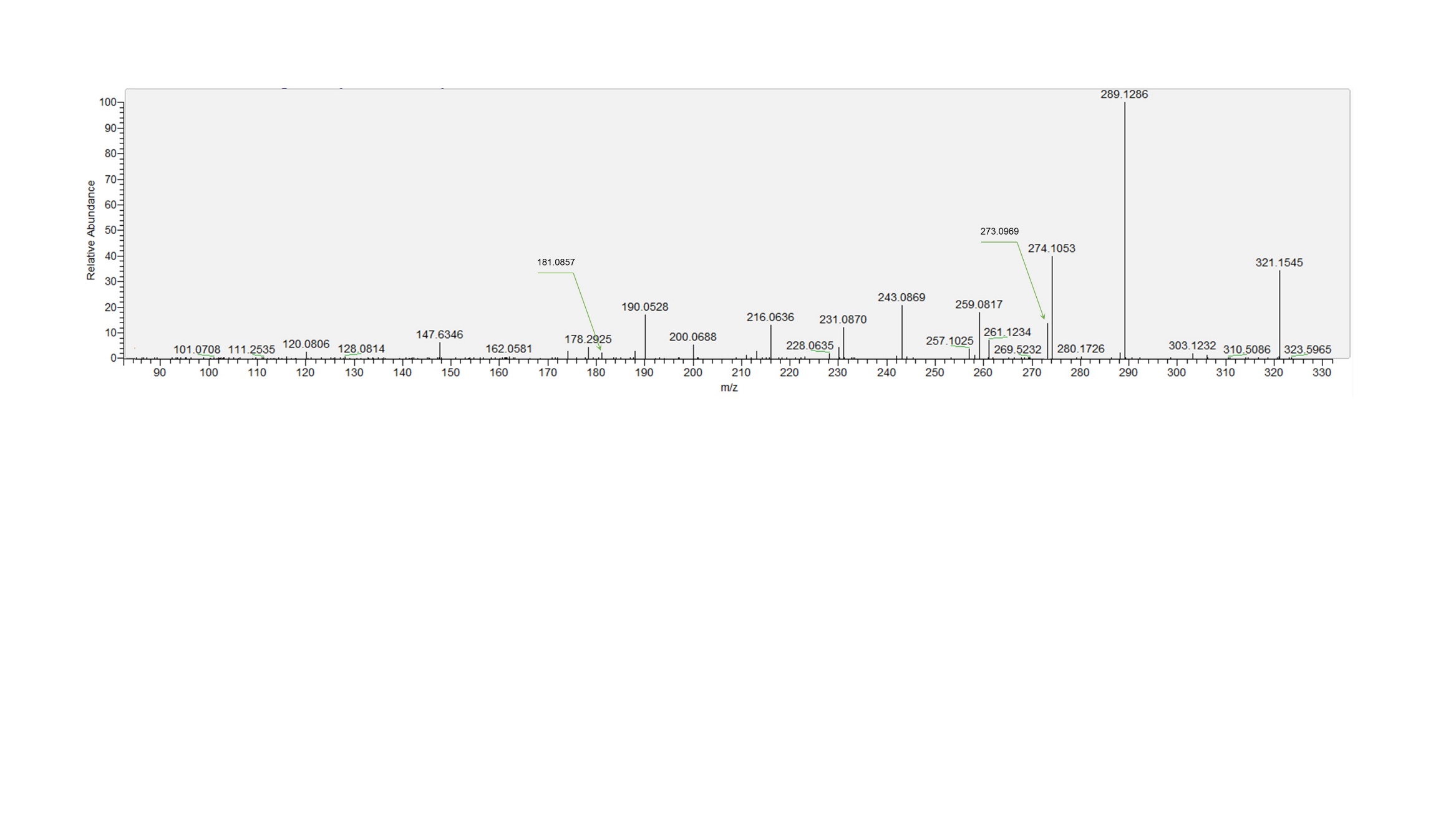


Figure S11. Mass spectrum of TMP321 (*N*-methyl-hydroxy TMP). (MS^2^ CID 60 eV)

The most dominant fragment in the TMP321 spectrum is 289.1286 ([C_14_H_16_N_4_O_3_+H]^+^, RDB= 8.5; error: -0.95 ppm), formed by loss of CH_2_O with the formation of a double bond. The fragmentation continues in keeping with the pattern of TMP, yielding: 274.1053 (-15.0233), 273.0969 (-16.0316), and 259.0817 (-30.0470).


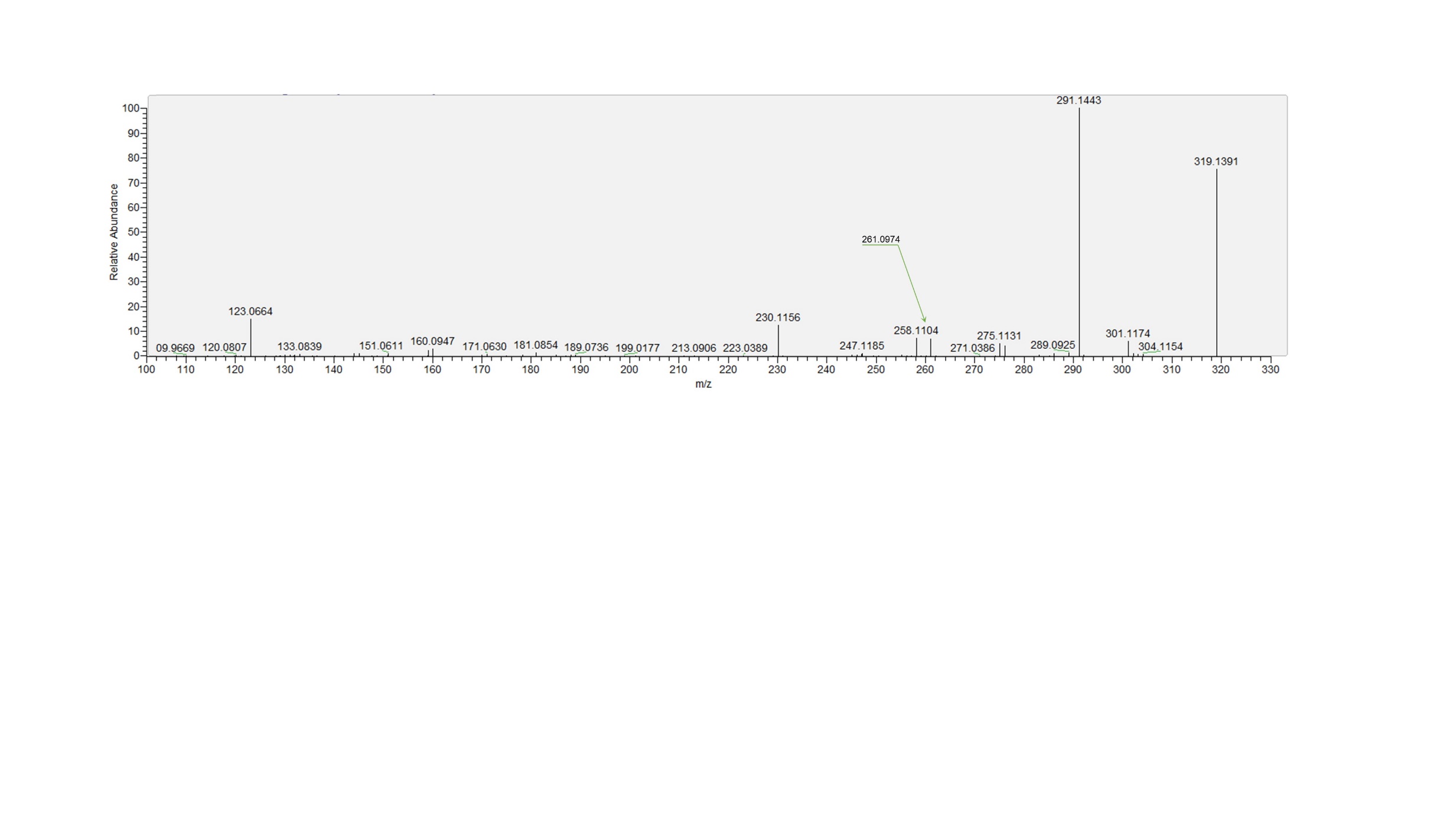


Figure S12. Mass spectrum of TMP319 (formyl TMP). (MS^2^ CID 60 eV)

The spectrum of TMP319 includes a base peak at 291.1443 that corresponds to TMP. Characteristic fragments of TMP can also be found, such as 275.1130, 261.0974, 230.1156, and 123.0664, which unambiguously confirm its presence.


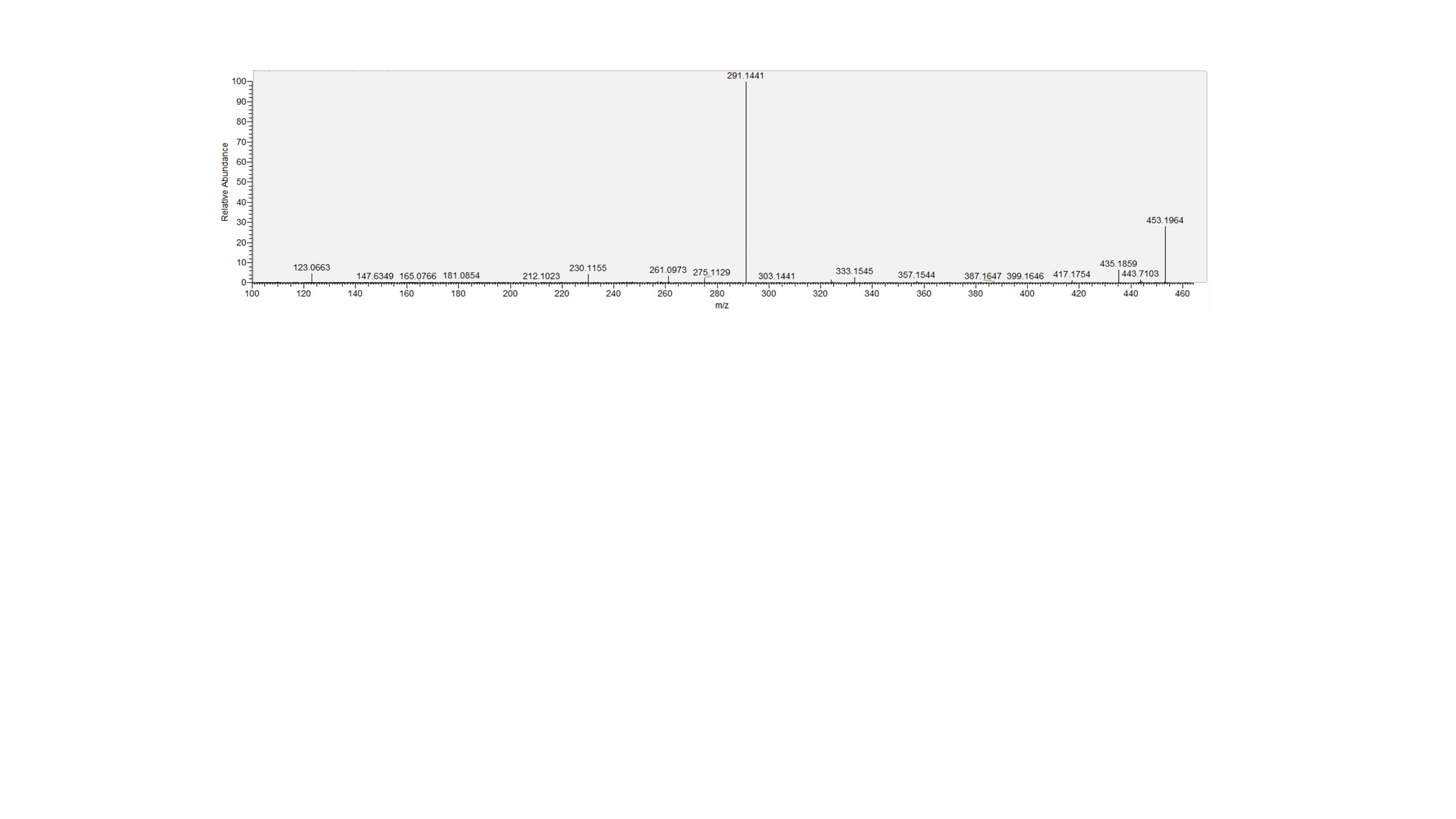
 Figure S13. Mass spectrum of TMP453 (*N*-glucorinated TMP). (MS^2^ CID 60 eV)

The major fragment observed in the spectra of TMP453, 291.1441 ([C_14_H_18_N_4_O_3_+H]^+^, RDB= 7.5, error= -3.56 ppm), confirmed that TMP453 incorporates the structure of TMP. Several minor fragments formed by water loss (characteristic for moieties with hydroxy groups) were also observed, such as 435.1859 ([C_20_H_26_N_4_O_7_+H]^+^, RDB= 9.5, error= -3.62 ppm) and 417.1754 ([C_20_H_24_N_4_O_6_+H]^+^, RDB= 10.5, error= -3.45 ppm), formed by sequential water loss. Additionally, 357.1544 ([C_18_H_20_N_4_O_4_+H]^+^, RDB= 10.5, error= -3.79 ppm) is produced by C_2_H_8_O_4_ loss from the parent metabolite.


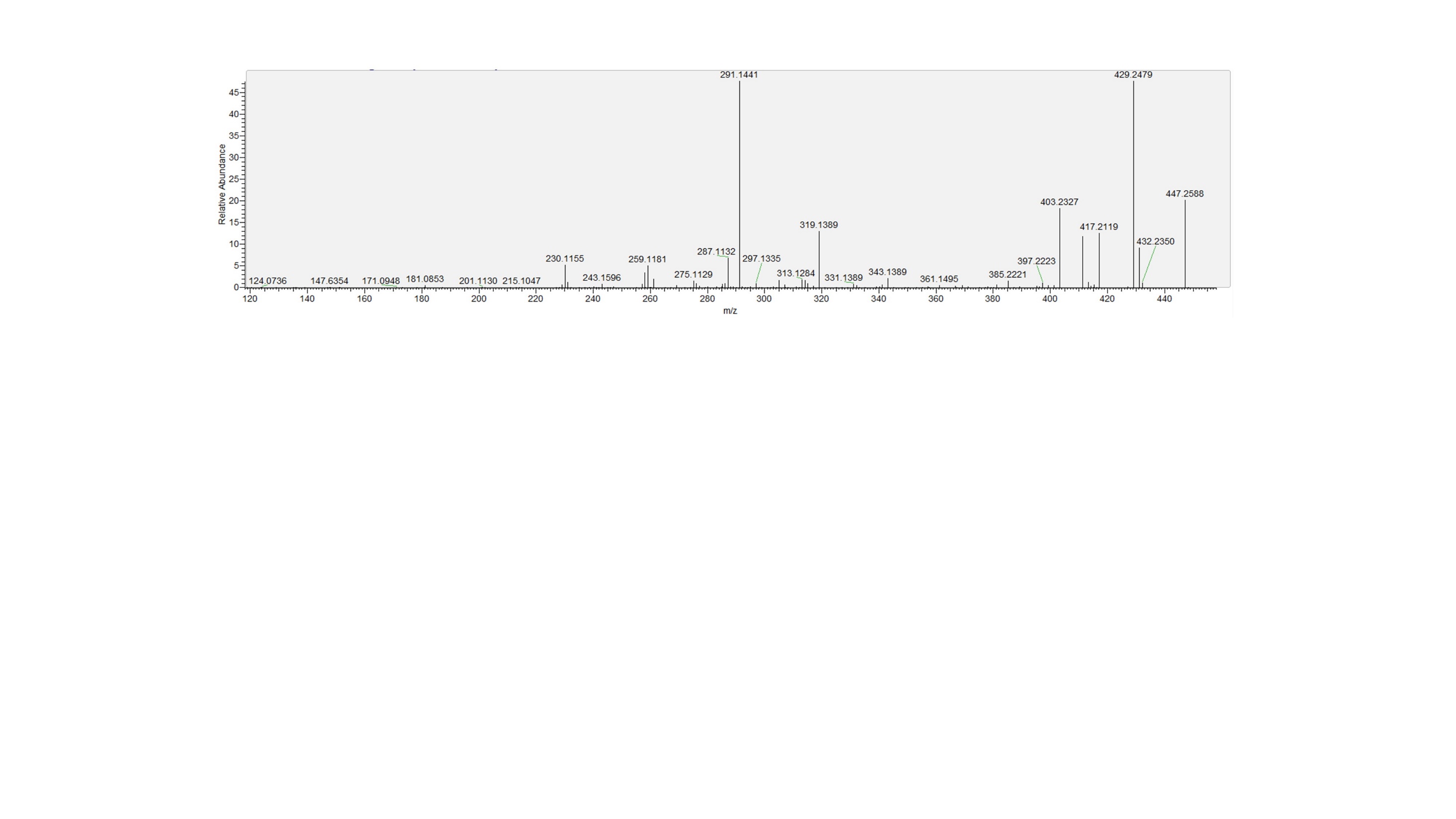


Figure S14. Mass spectrum of TMP447 (*N*-8-hydroxynonanoic acid TMP). (MS^2^ CID 60 eV)


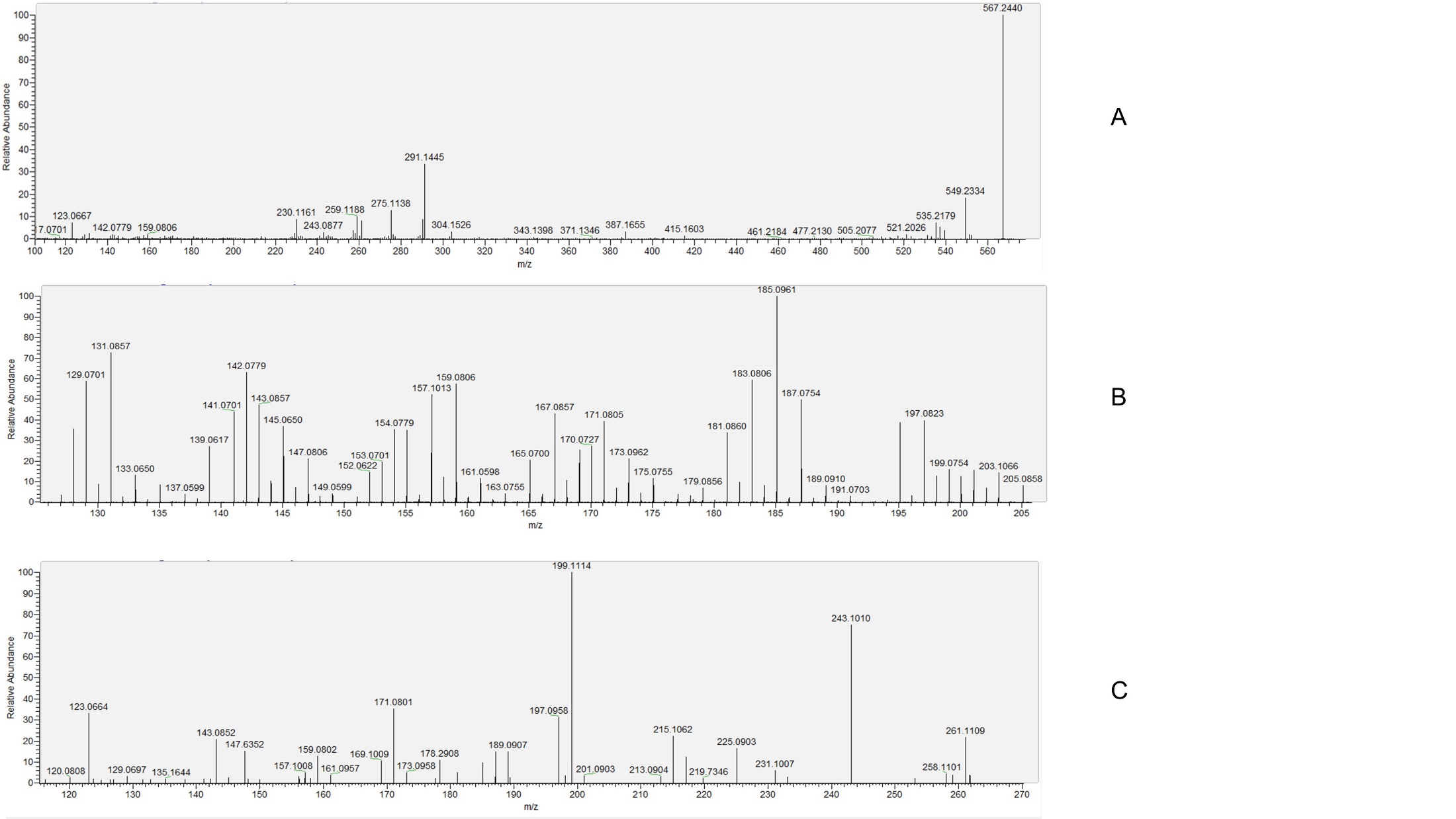


Figure S15. Mass spectrum of TMP567. A – MS^2^ CID 60eV; B – zoomed MS^2^ CID 60 eV; C –MS^3^ 567.2440 > 549.2334 CID 60eV


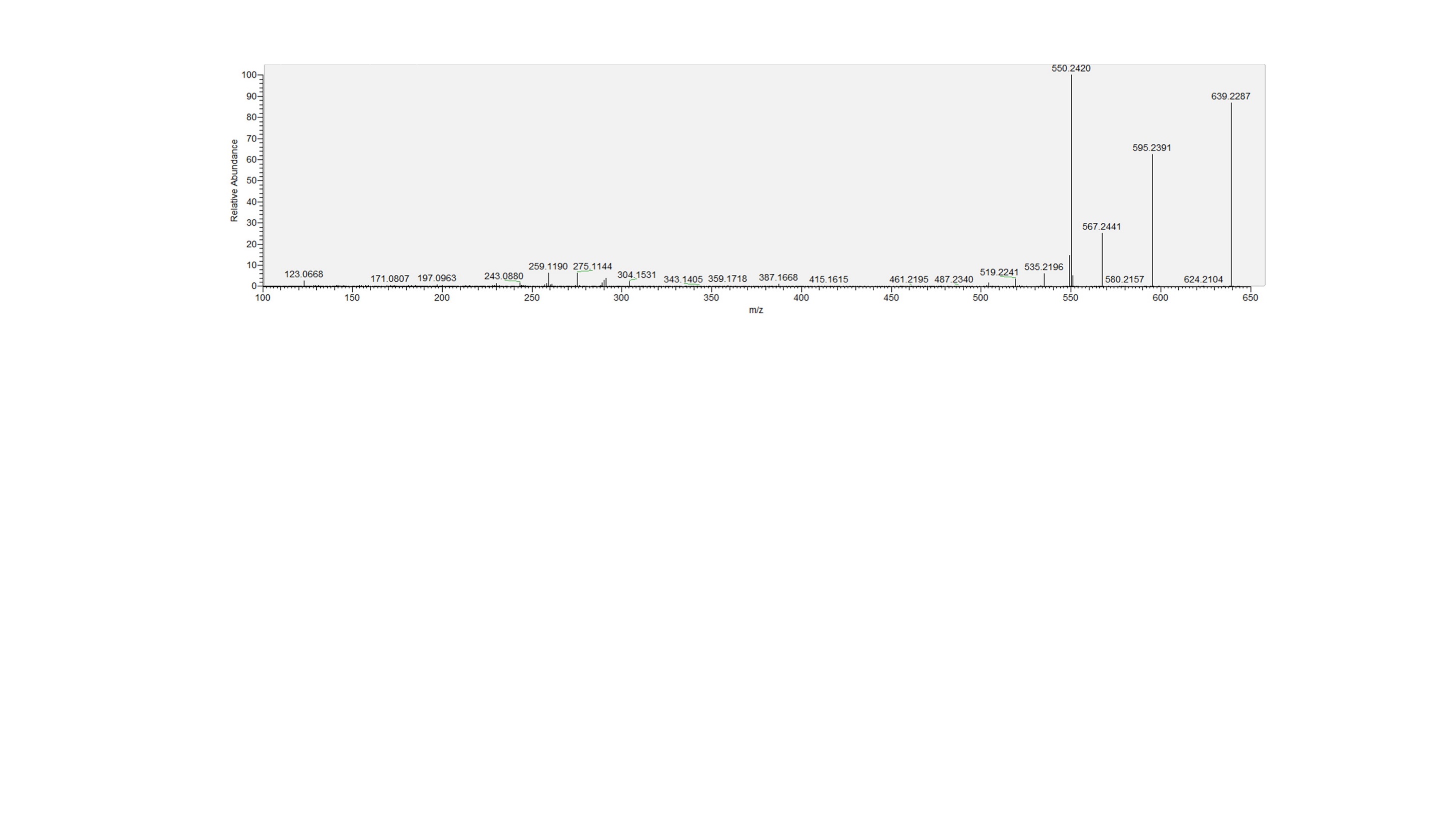
 Figure S16. Mass spectrum of TMP639 (MS^2^ CID 60 eV)


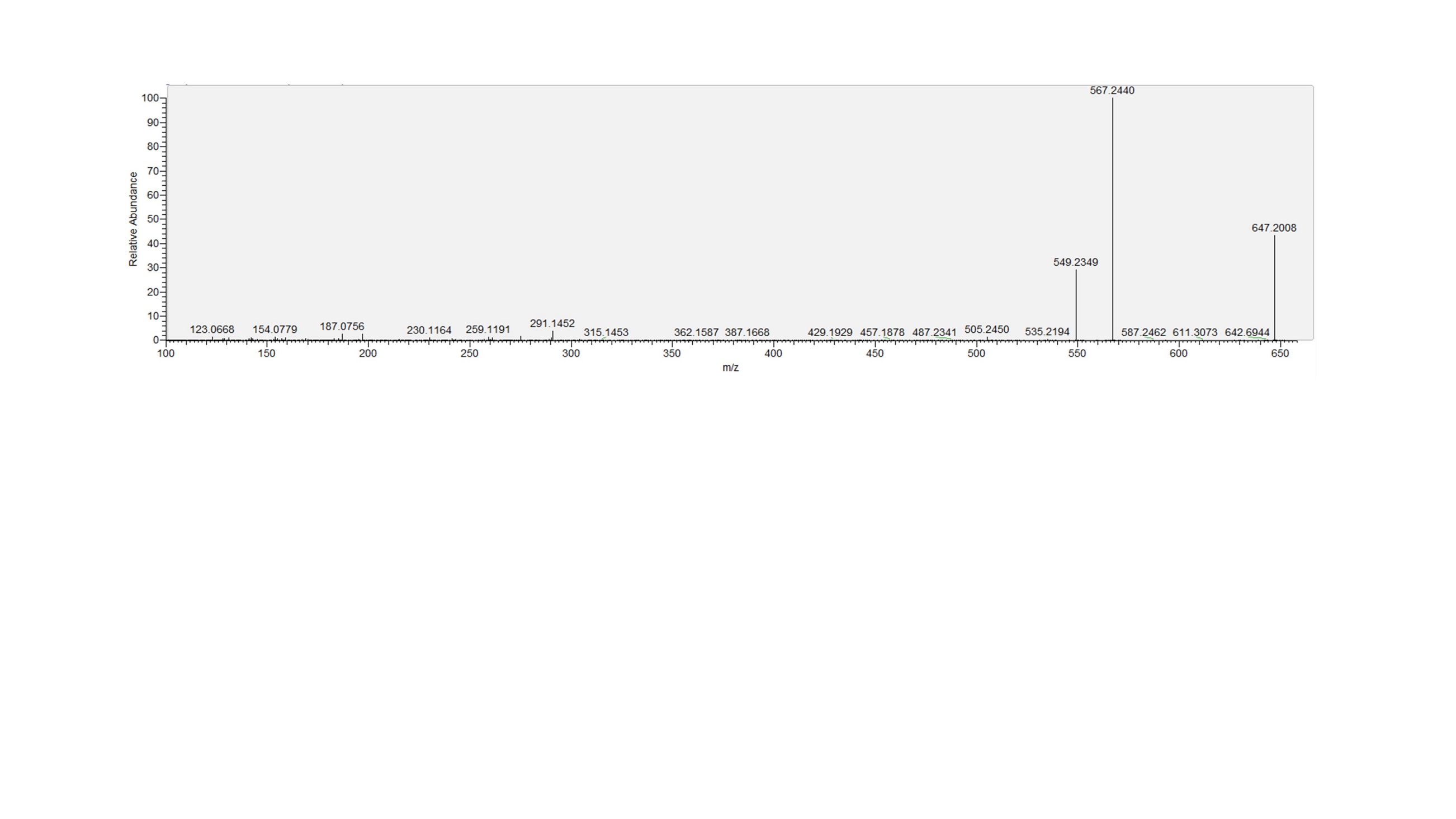


Figure S17. Mass spectrum of TMP647 (MS^2^ CID 60 eV)


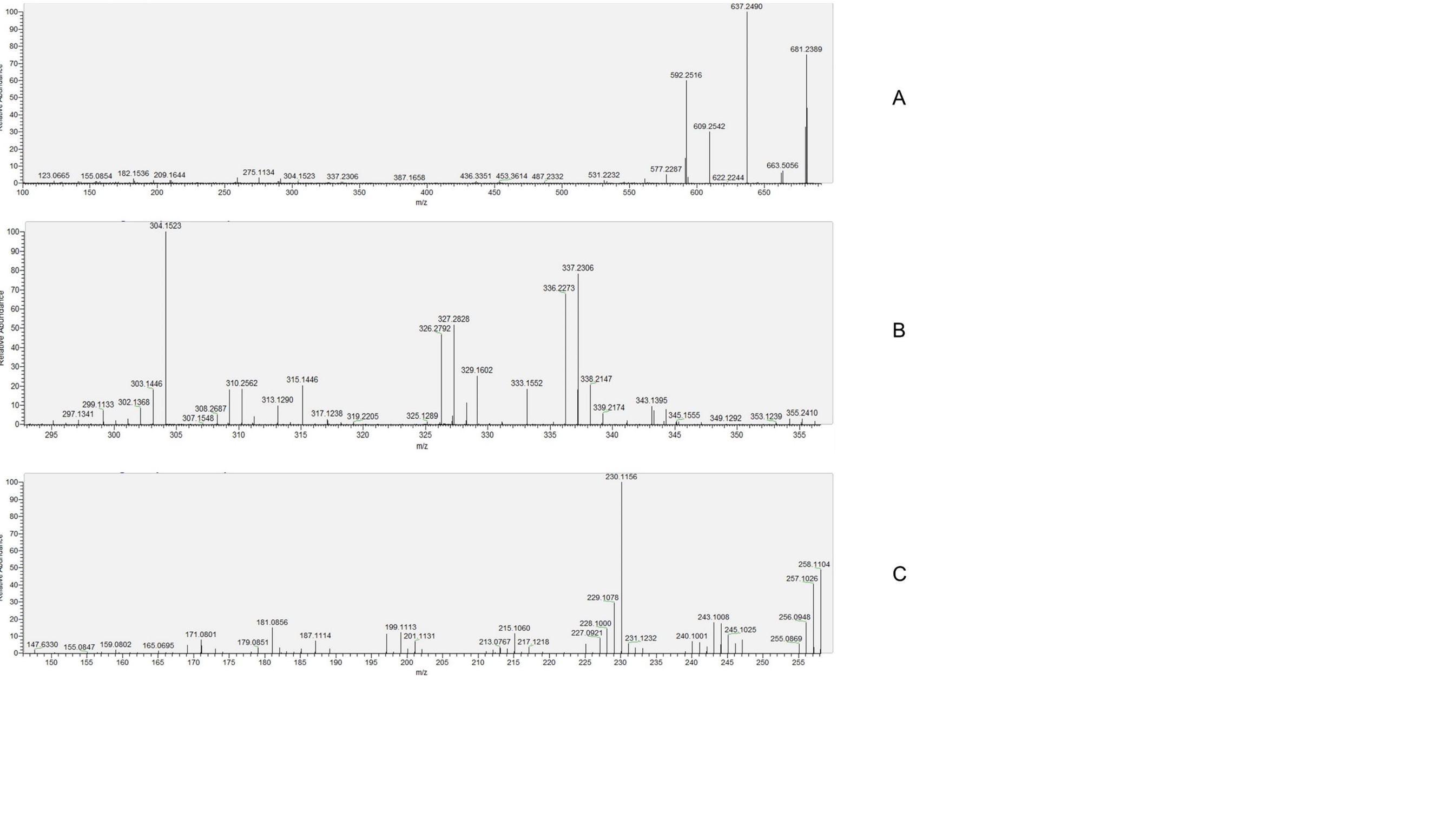


Figure S18. Mass spectrum of TMP681. A – MS^2^ CID 60eV; B and C - zoomed MS^2^ CID 60 eV


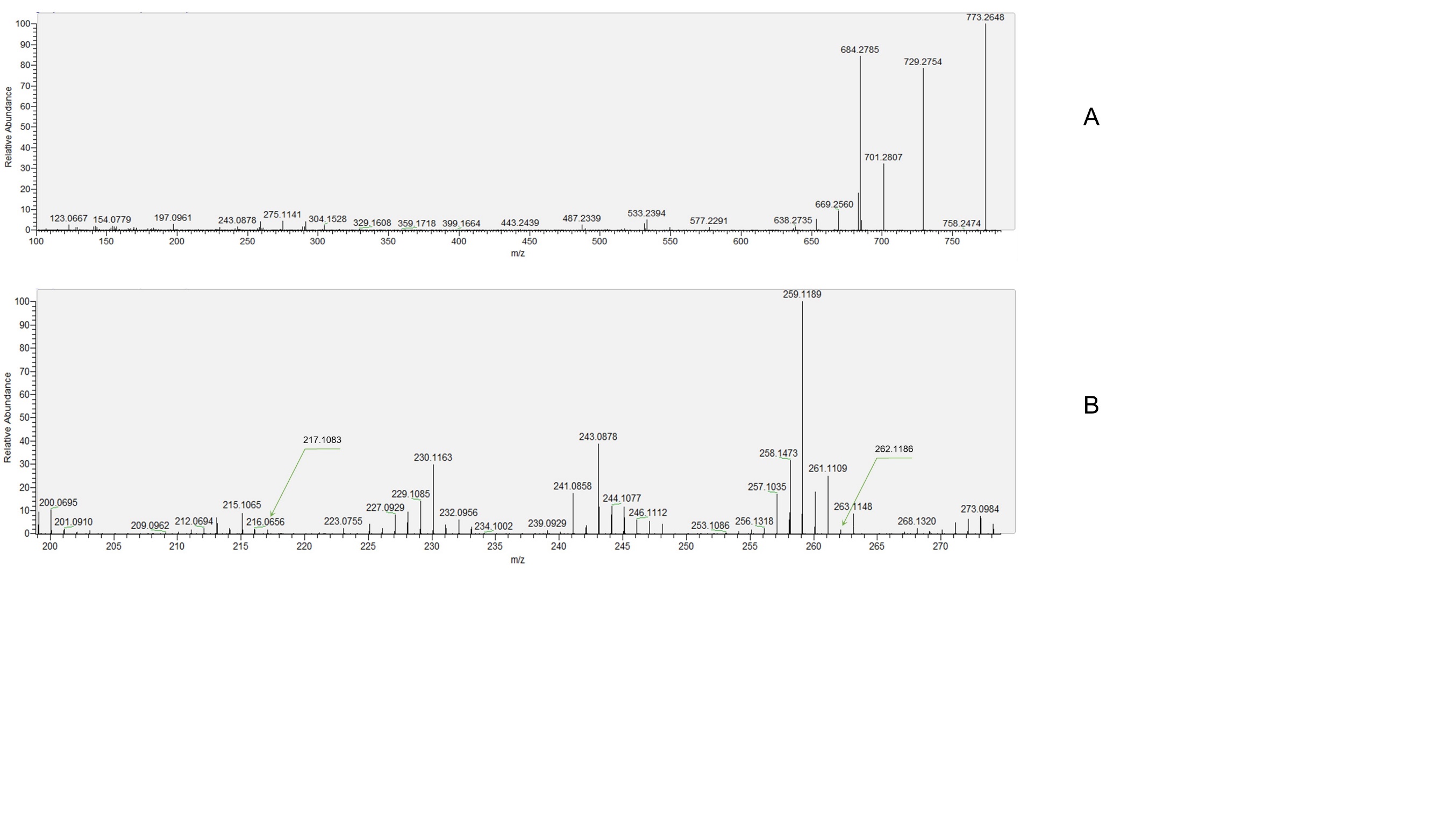
Figure S19. Average CID mass spectrum of TMP773. A – MS^2^ CID 60eV; B - zoomed MS^2^ CID 60 eV


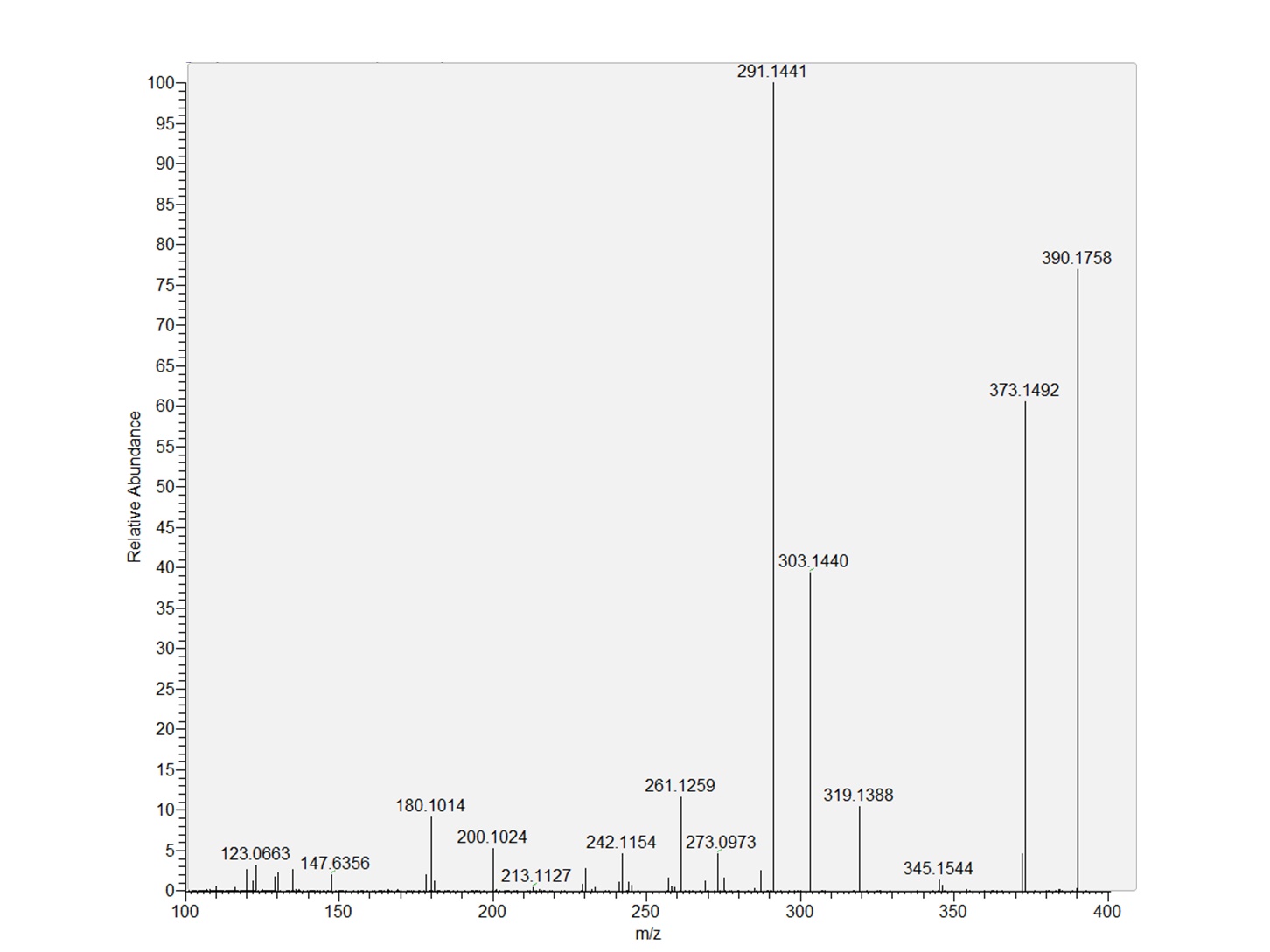


Figure S20. Average CID mass spectrum of TMP390

In the mass spectrum of TMP390 ([C_18_H_23_N_5_O_5_+H]^+^, RDB= 9.5, error= -1.38), formation of 373.1492 ([C_18_H_20_N_4_O_5_+H]^+^, RDB= 10.5, error= -3.87 ppm) happened as a result of a neutral loss of NH_3_ with the formation of a double bond, which further yielded 345.1544 ([C_17_H_20_N_4_O_4_+H]^+^, RDB= 9.5, error= -3.86 ppm) through neutral loss of CO. The aforementioned fragments indicate the occurrence of NH_2_ and OH groups. Fragmentation continues with further loss of C_2_H_2_ (-26.0155) and formation of 319.1388 ([C_15_H_18_N_4_O_4_+H]^+^, RDB= 8.5, error= -3.98 ppm), which, in fact, is formyl TMP, which, through the release of an oxygen atom, formed 303.1444 ([C_15_H_18_N_4_O_3_+H]^+^, RDB= 8.5, error= -3.71 ppm). In addition, 291.1441 confirms the occurrence of the TMP scaffold in the structure of this metabolite. This suggests that L-aspartate 4-semialdehyde could be a molecule conjugated with TMP.


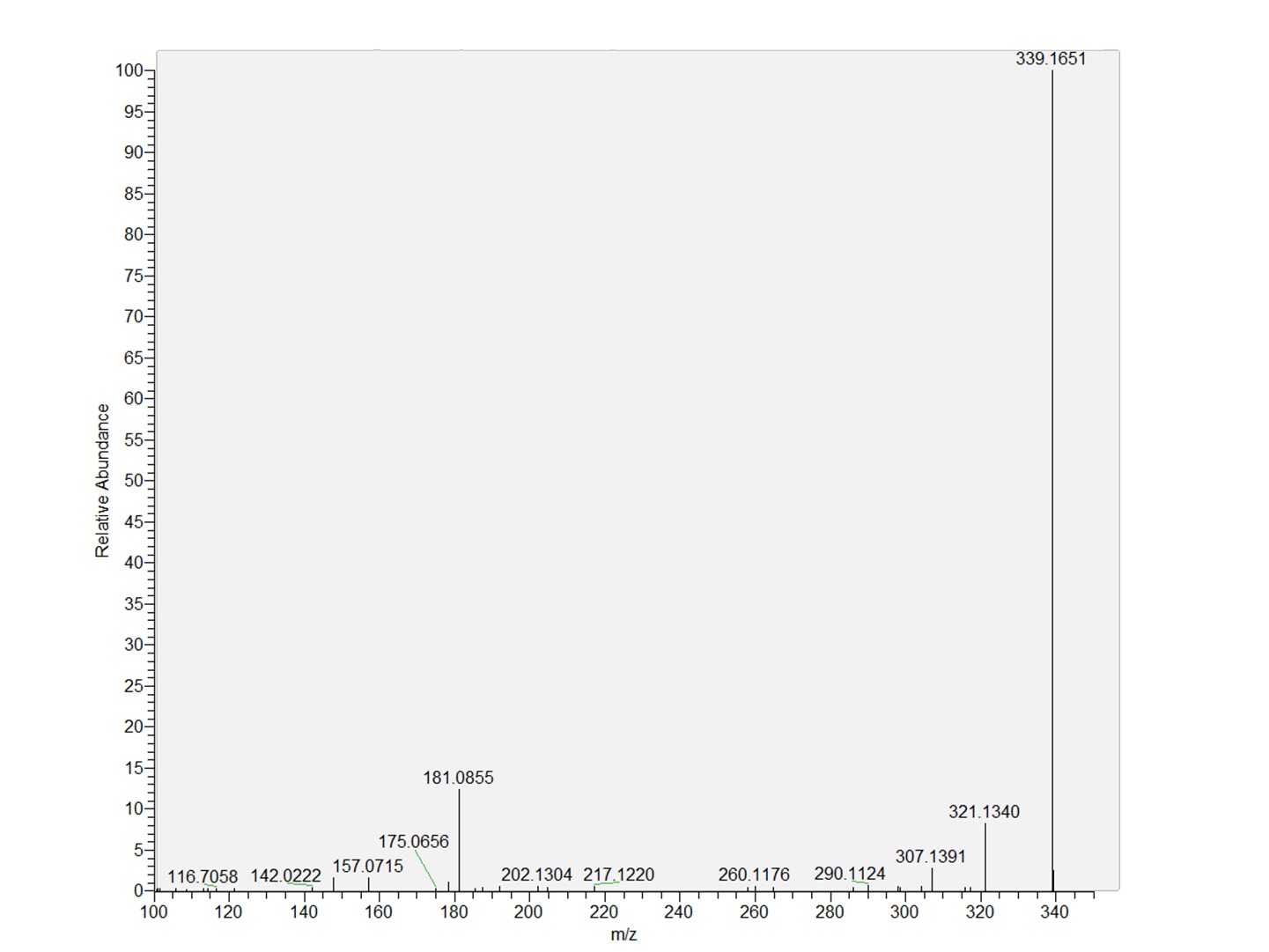


Figure S21. Average CID mass spectrum of TMP339

An estimated difference between TMP339 ([C_15_H_22_N_4_O_5_+H]^+^, RDB= 6.5, error= -3.44 ppm) and TMP accounts for two additional oxygen atoms and a methyl group. Fragment 181.0855 indicates that the 1,2,3-trimethoxybenzene moiety is intact, making it possible to conclude that the metabolisation took place at the 2,4-diaminopyrimidine. This may suggest that methylation of the amino group and double hydroxylation (on the 2,4-diaminopyrimidine and methylene bridge) with hydrogenation are the biotransformation reactions that led to the formation of TMP339.


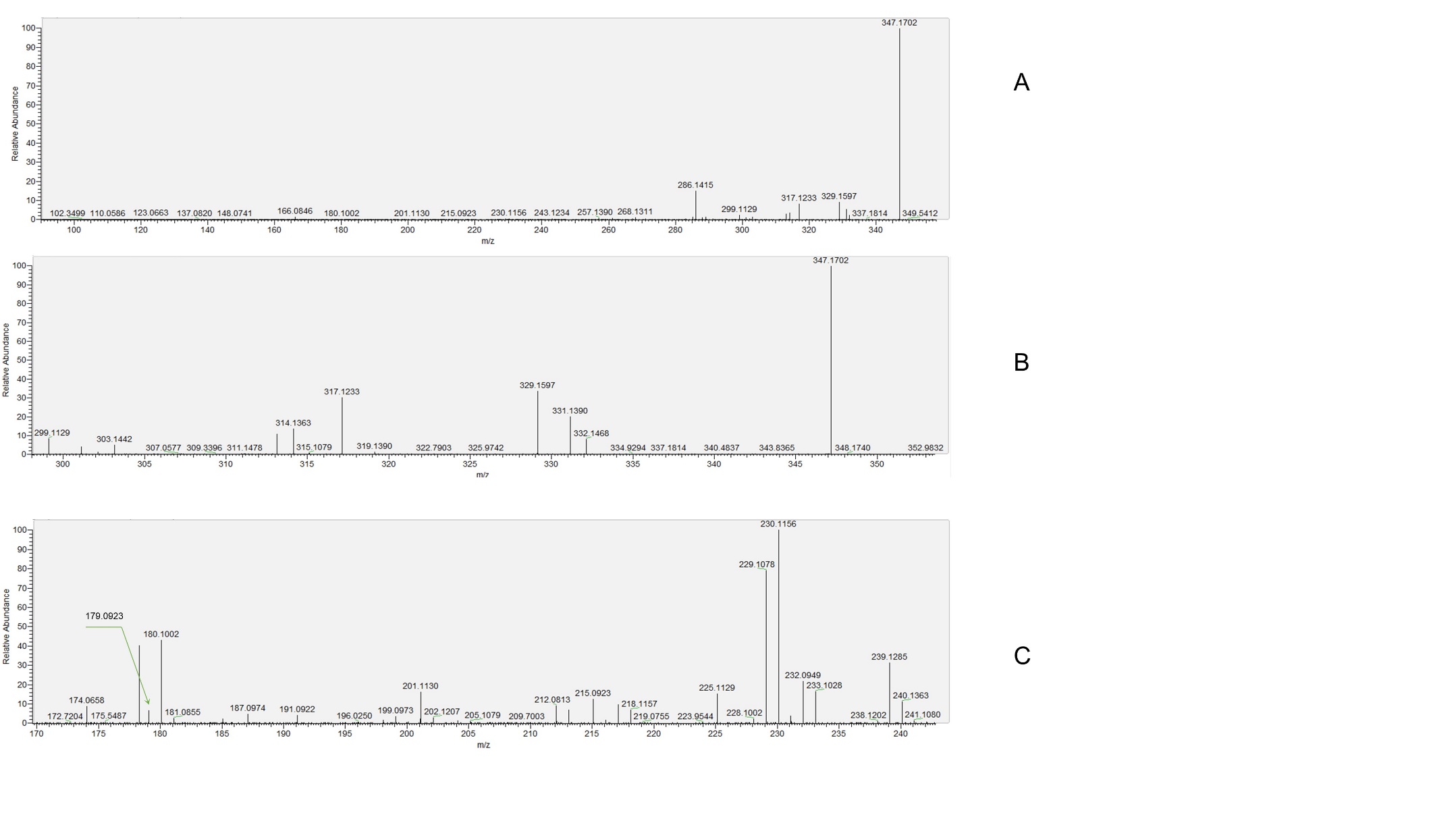


Figure S22. Mmass spectrum of TMP347. A - MS^2^ CID 60eV; B - MS^2^ HCD 40eV; C zoomed MS^2^ CID 60eV

The TMP347 spectrum ([C_17_H_22_N_4_O_4_+H]^+^, RDB= 8.5, error= -3.46 ppm) revealed the typical fragmentation pattern for TMP, specifically, the following m/z values: 332.1468 (-15.0234), 331.1390 (-16.0312), 317.1233 (-30.0468), and 314.1363 (-33.0338). The difference between TMP347 and TMP of 56.0260 corresponds to the addition of C_3_H_4_O. The occurrence of 179.0923 ([C_8_H_10_N_4_O+H]^+^, RDB= 5.5, error= -2.55 ppm) and the 239.1285 ([C_13_H_18_O_4_+H]^+^, RDB= 4.5, error= 2.74 ppm) indicate that the conjugation took place between the rings on the methylene bridge (α-position). An added molecule that could fit this description could be lactaldehyde.


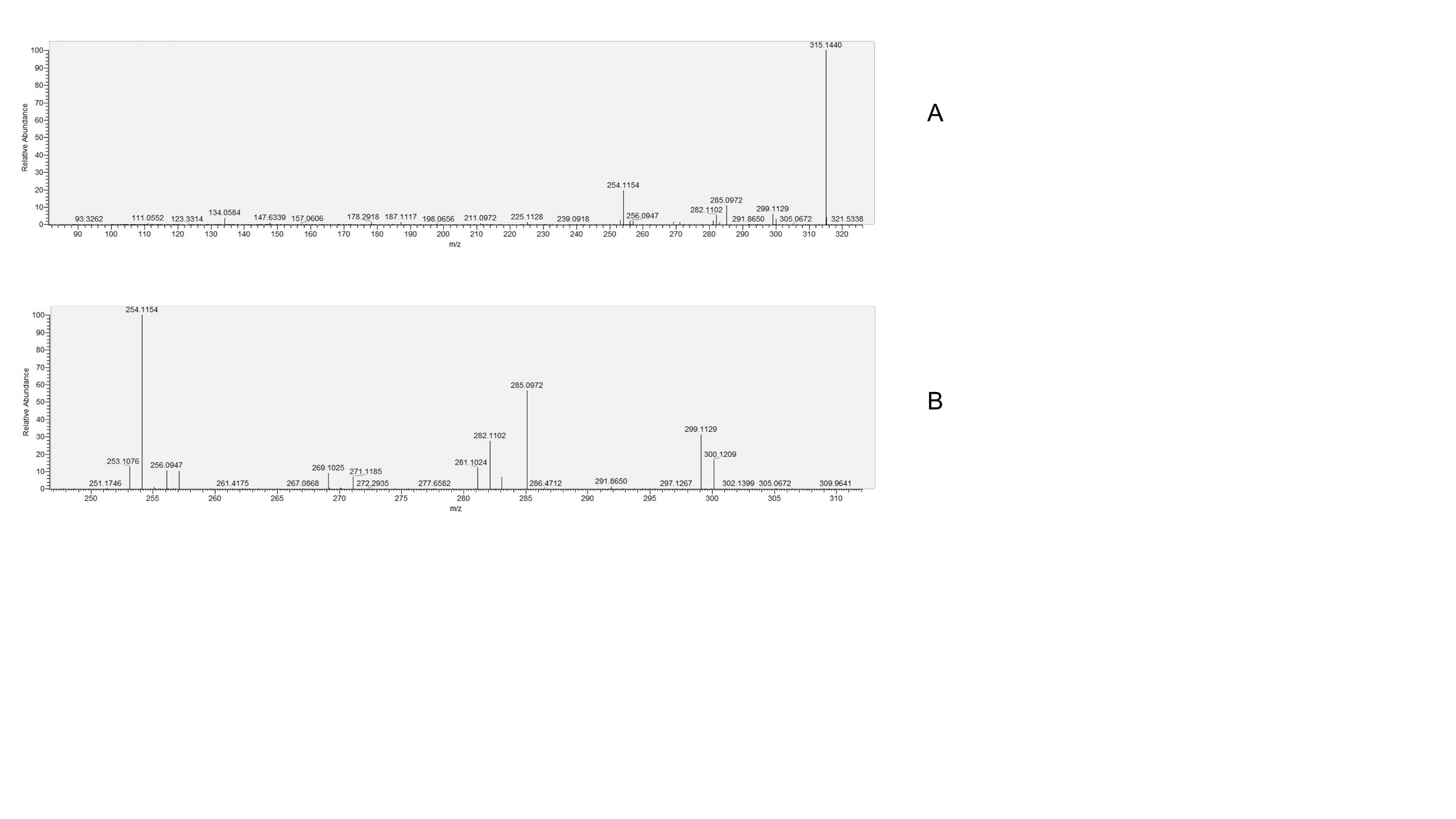


Figure S23. Average CID mass spectrum of TMP315. A - MS^2^ CID 60eV; B - zoomed MS^2^ CID 60eV

An estimated difference (24.001 m/z) between TMP315 ([C_16_H_18_N_4_O_3_+H]^+^, RDB= 9.5, error= -3.57 ppm) and TMP accounts for two carbon atoms and could indicate the addition of C_2_H_2_ plus double bond formation. Metabolization happened on the pyrimidine, as evidenced by observed fragment 134.0584 (110.0587 + 24.001). There is a lack of fragments that would indicate another position on the TMP scaffold. A typical fragmentation pattern for TMP was observed for this metabolite as well, generating the following fragments: 300.1209 (-15.0232), 299.1129 (-16.0312), 285.0972 (-30.0468), 282.1102 (-33.0338), and 254.1154 (-61.0286).


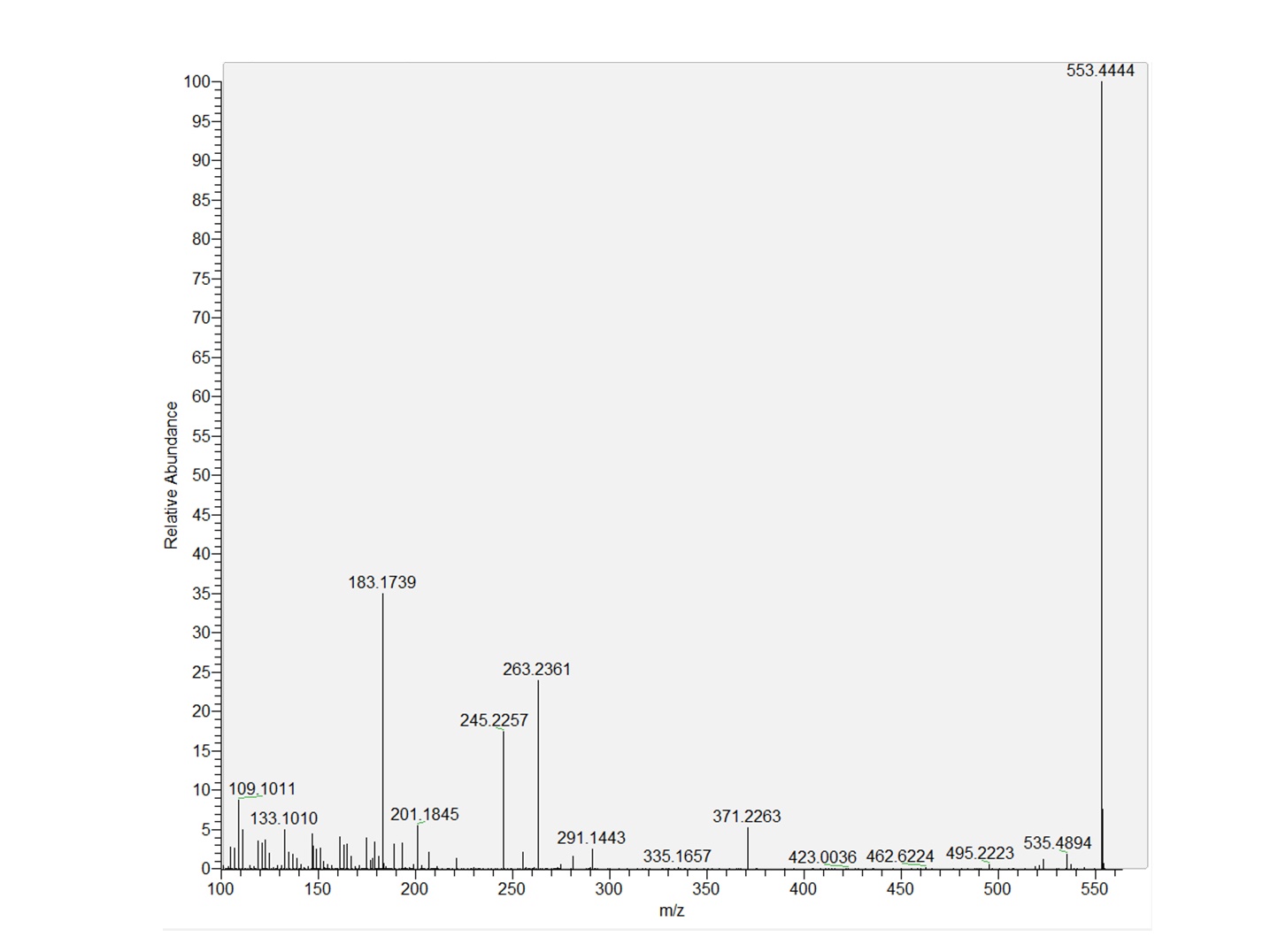


Figure S24. Average CID mass spectrum of TMP553_a


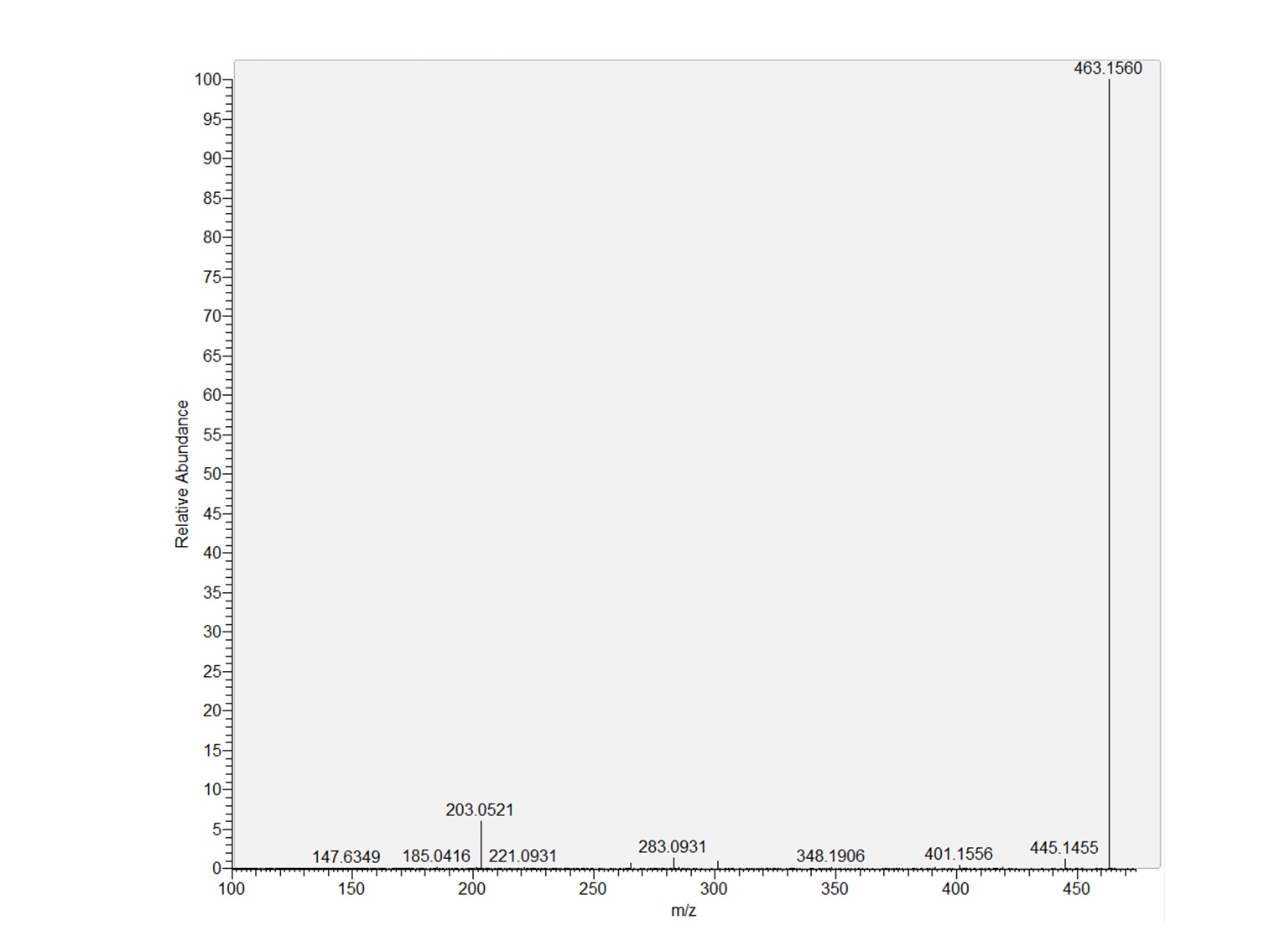


Figure S25. Average CID mass spectrum of TMP463


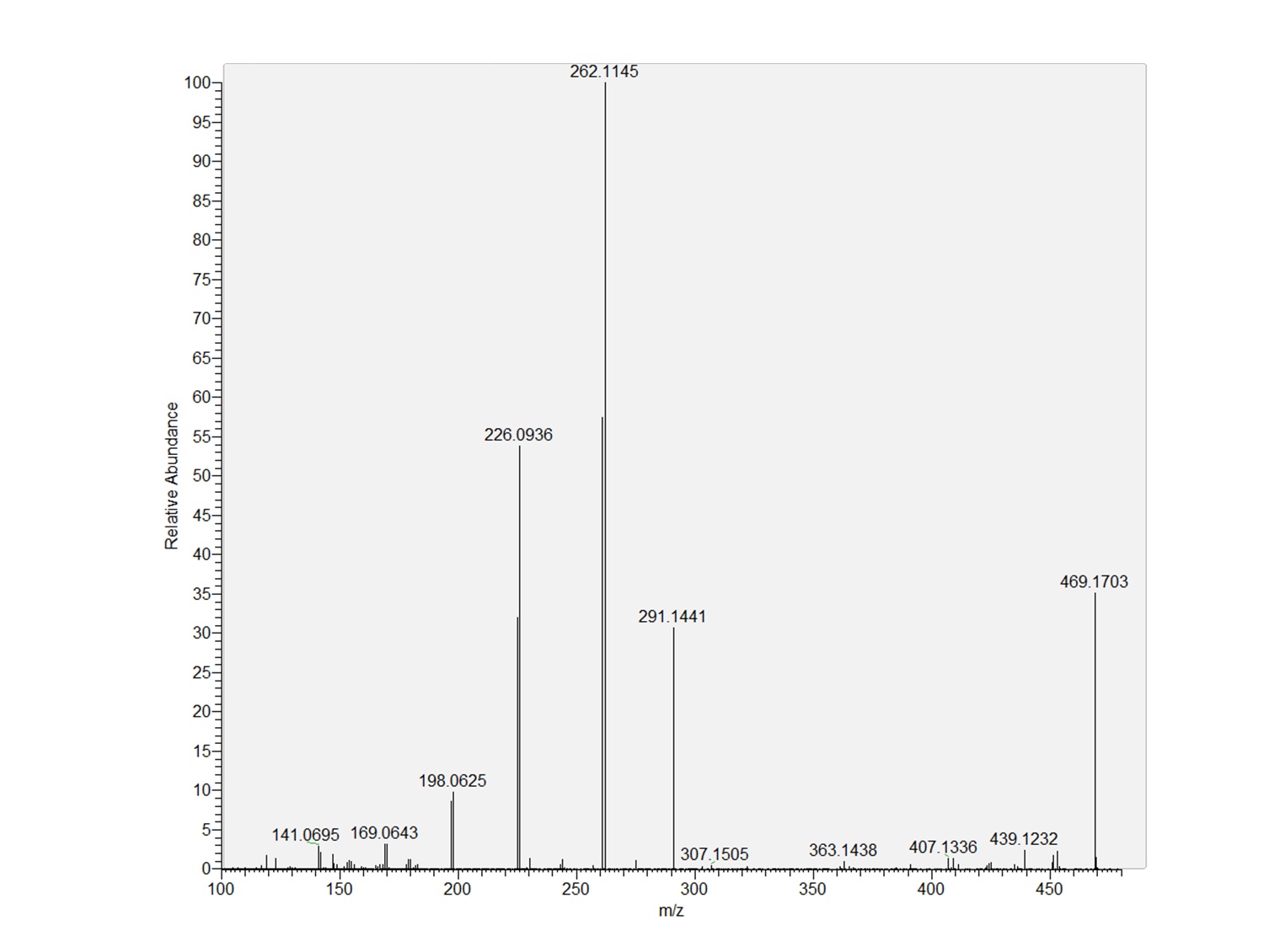


Figure S26. Average CID mass spectrum of TMP469


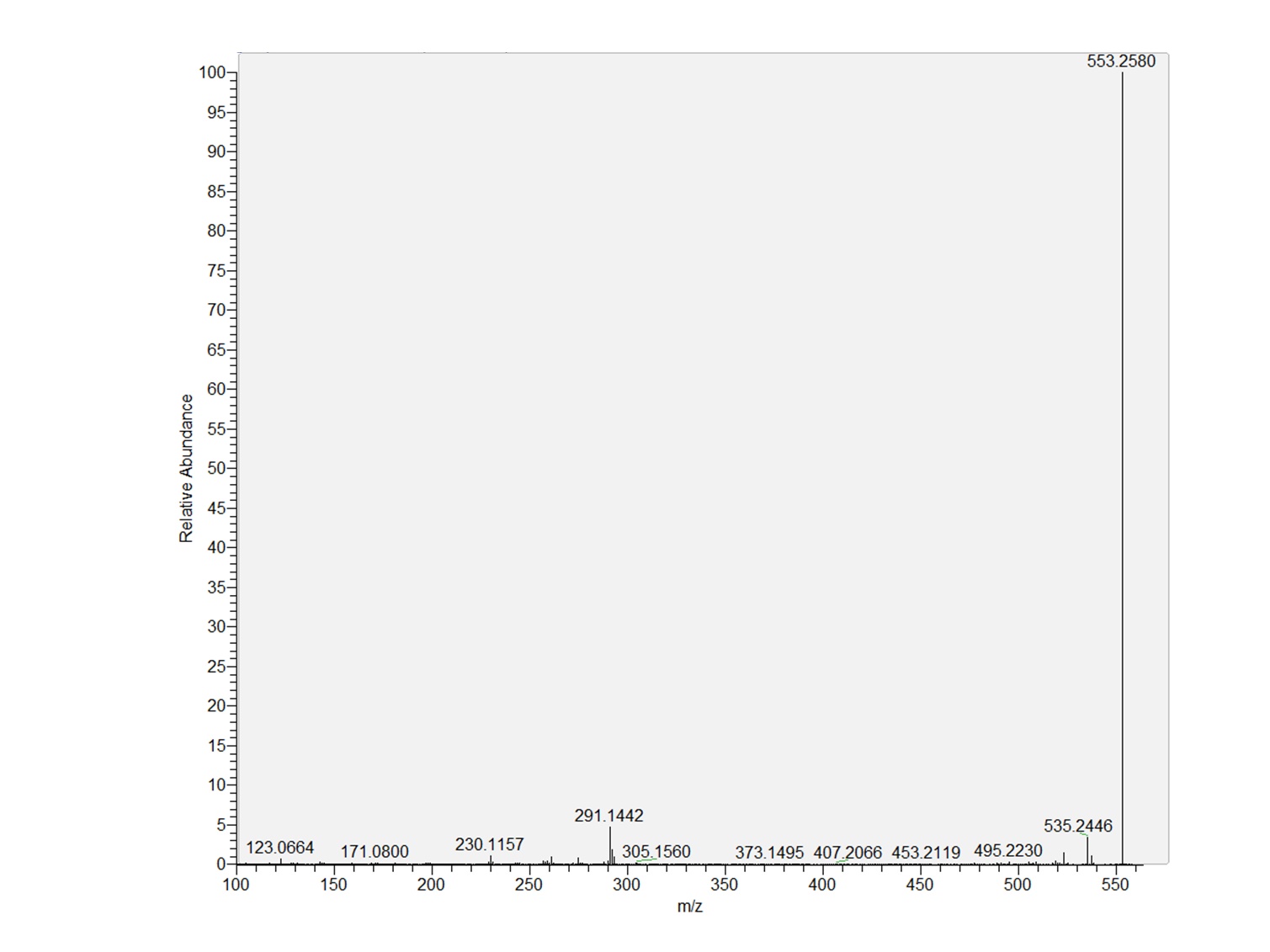


Figure S27. Average CID mass spectrum of TMP553_b


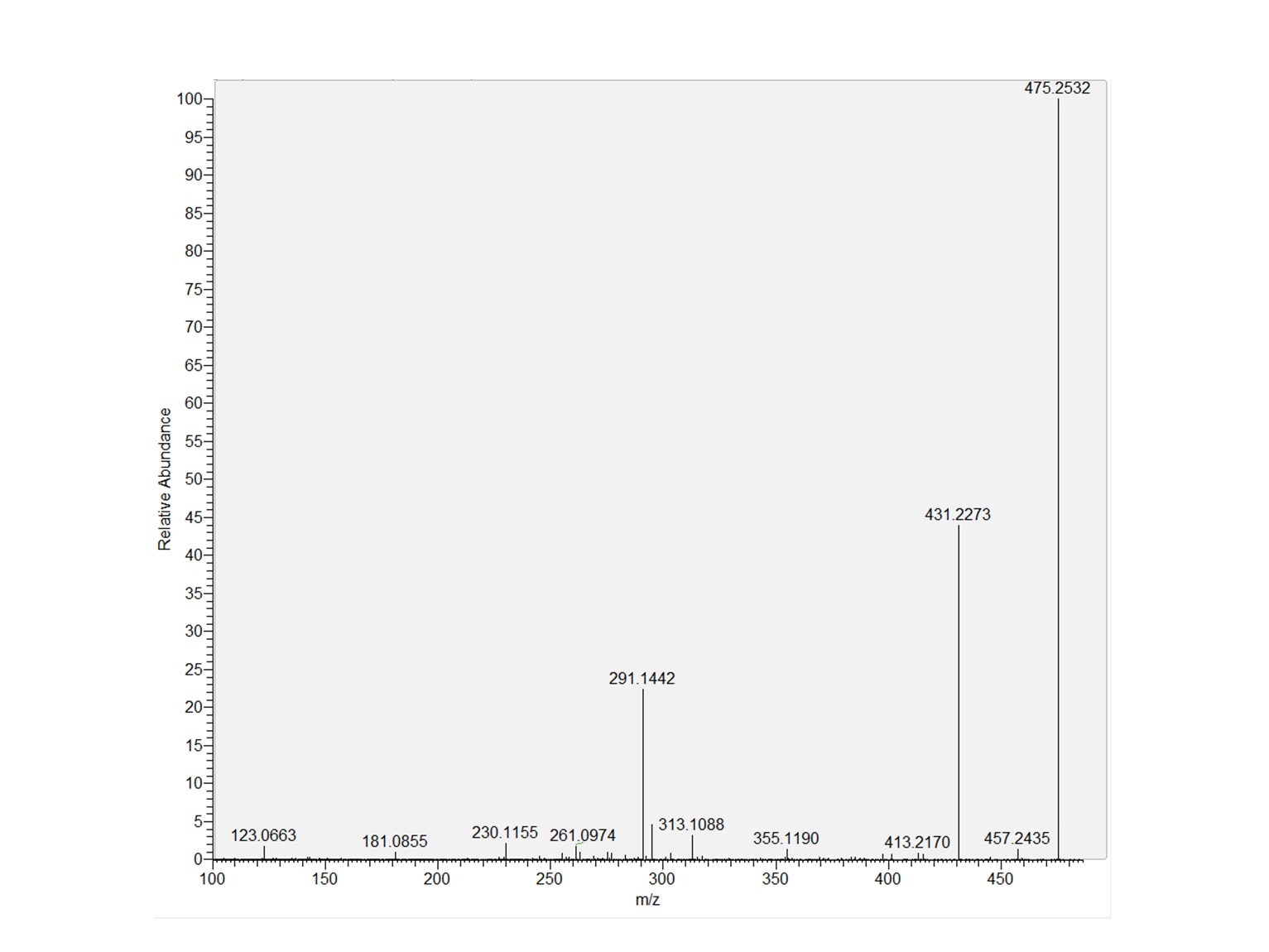


Figure S28. Average CID mass spectrum of TMP475


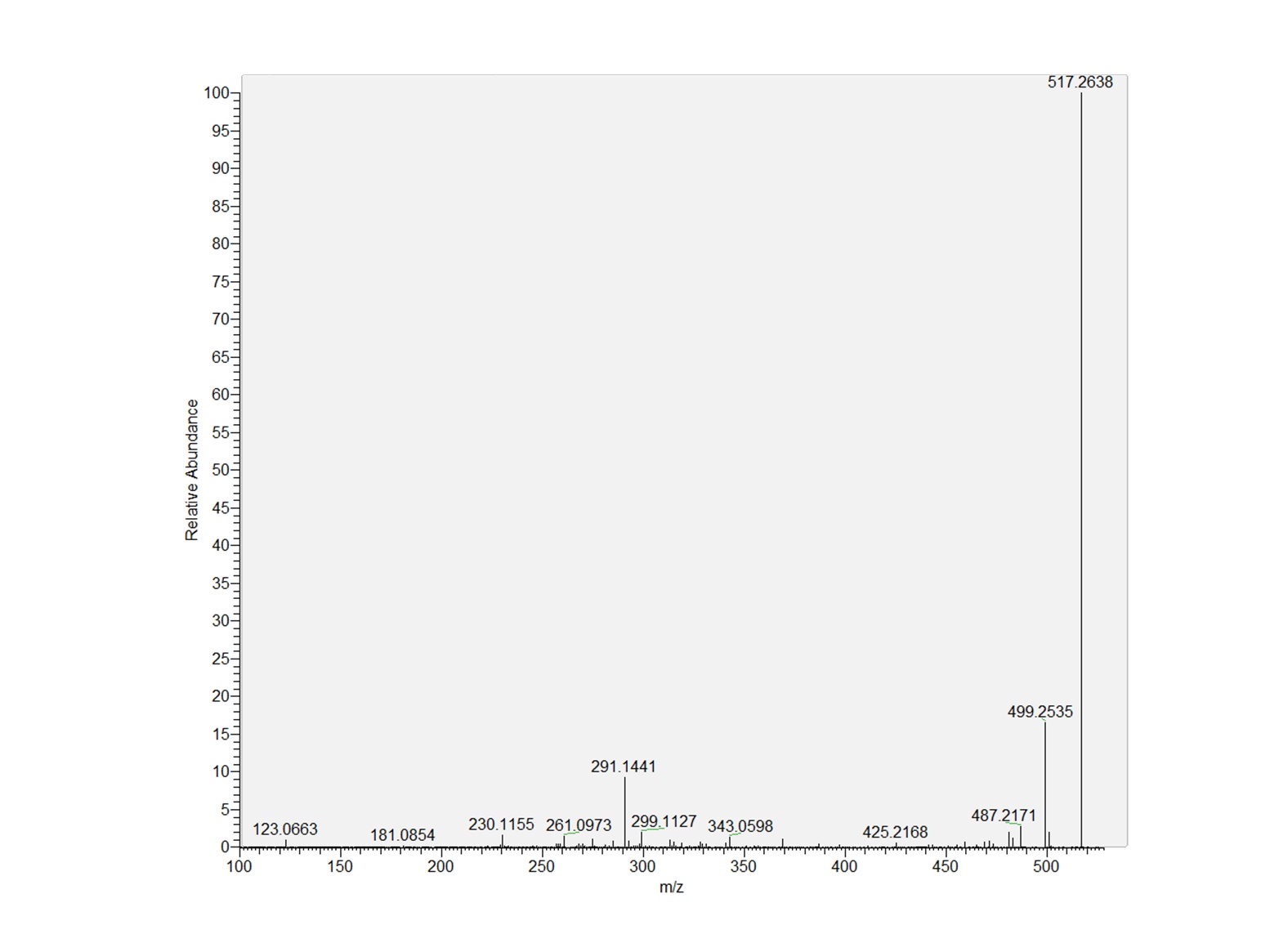


Figure S29. Average CID mass spectrum of TMP517


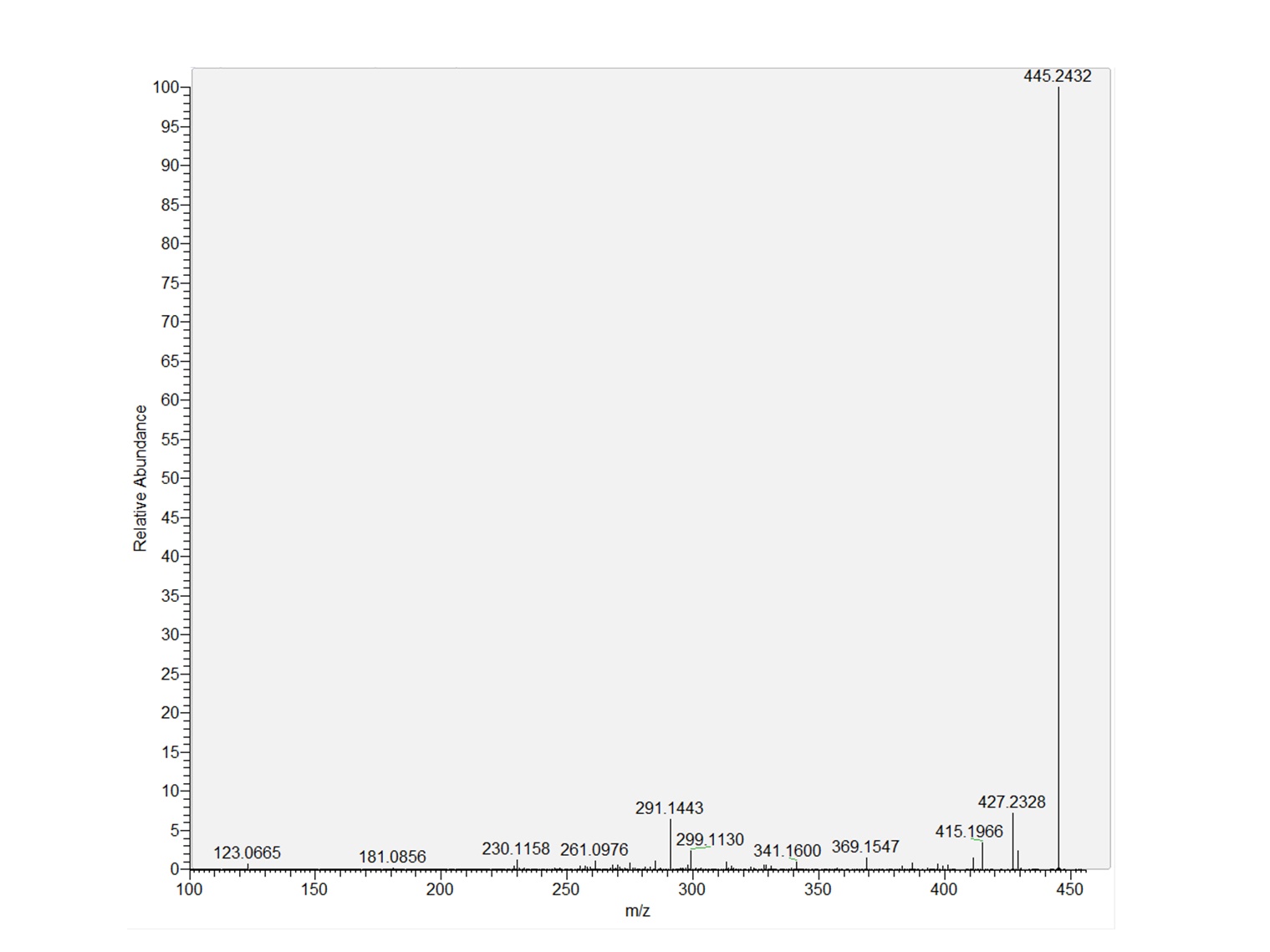


Figure S30. Average CID mass spectrum of TMP445

Table S1. Estimated solubility, acute and chronic toxicity values for fish, daphnid and green algae of TMP and its metabolites

| Compounds | Solubility ^a^ (mg/L) | Acute toxicity (mg L^-1^) | | | Chronic toxicity (mg L^-1^) | | |
| --- | --- | --- | --- | --- | --- | --- | --- |
|  |  | Fish  (96 hrs, LC_50_ ^b^) | Daphnid  (48 hrs, LC_50_ ^b^) | Green algae  (96 hrs, EC_50_ ^c^) | Fish  (ChV ^d^) | Daphnid  (ChV ^d^) | Green algae  (ChV ^d^) |
| TMP307 | 5.53e4 | 69451 | 4806 | 11626 | 21202 | 232 | 2608 |
| TMP277 | 8127 | 10245 | 814 | 1496 | 2046 | 45 | 371 |
| TMP261 | 3077 | 523 | 52 | 61 | 52 | 3.6 | 17 |
| TMP453 | 3019 | 4689 | 1701 | 5512 | 541 | 83 | 544 |
| TMP325 | 1e6 | 188374 | 12128 | 33847 | 71684 | 547 | 7207 |
| TMP349 | 1659 | 6842 | 2573 | 9118 | 945 | 124 | 761 |
| TMP | 2324 | 211 | 6.4 | 20 | 3.6 | 0.1 | 9.6 |
| TMP321 | 1.56e4 | 2842 | 1022 | 3241 | 314 | 50 | 333 |
| TMP305 | 4870 | 342 | 7.9 | 27 | 6.5 | 0.1 | 14 |
| TMP319 | 2064 | 689 | 229 | 596 | 51 | 11 | 88 |
| TMP567 | 52 | 369 | 871 | 446 | 35 | 836 | 78 |
| TMP639 | 154 | 138 | 298 | 134 | 11 | 226 | 30 |
| TMP551 | 7.2 | 78 | 163 | 70 | 5.9 | 113 | 17 |
| TMP647 | 3544 | 17121 | 54227 | 43032 | 2941 | 114900 | 3076 |
| TMP681 | 1.1 | 105 | 221 | 95 | 8.1 | 156 | 23 |
| TMP447 | 9.7 | 85 | 24 | 46 | 3.2 | 1.3 | 12 |
| TMP537 | 1.2 | 60 | 17 | 29 | 1.9 | 0.89 | 9.5 |
| TMP773 | 0.6 | 112 | 234 | 100 | 8.5 | 163 | 25 |
| TMP549 | 7.5 | 14 | 26 | 9.3 | 0.84 | 12 | 3.5 |
| TMP631 | 219 | 2110 | 5668 | 3525 | 262 | 7716 | 415 |

^a^ water solubility at 25 °C estimated by EPIsuite; ^b^ LC_50_ (lethal concentration for 50% kill); ^c^ EC_50_ (concentration for 50% effectiveness); ^d^ ChV (chronic value)
